# Fluid Relaxation Approximation of the Busenberg–Travis Cross-Diffusion System

**DOI:** 10.1007/s00220-025-05341-2

**Published:** 2025-06-03

**Authors:** José Antonio Carrillo, Xiuqing Chen, Bang Du, Ansgar Jüngel

**Affiliations:** 1 Mathematical Institute, University of Oxford, Oxford, OX2 66G UK; 2 School of Mathematics (Zhuhai), Sun Yat-sen University, Zhuhai, 519082 Guangdong Province China; 3 Institute of Analysis and Scientific Computing, TU Wien, Wiedner Hauptstrasse 8–10, 1040 Wien, Austria

## Abstract

The Busenberg–Travis cross-diffusion system for segregating populations is approximated by the compressible Navier–Stokes–Korteweg equations on the torus, including a density-dependent viscosity and drag forces. The Korteweg term can be associated to the quantum Bohm potential. The singular asymptotic limit is proved rigorously using compactness and relative entropy methods. The novelty is the derivation of energy and entropy inequalities, which reduce in the asymptotic limit to the Boltzmann–Shannon and Rao entropy inequalities, thus revealing the double entropy structure of the limiting Busenberg–Travis system.

## Introduction

The aim of this paper is to analyze a fluiddynamical approximation of the Busenberg–Travis population cross-diffusion system [7]

1 $$\begin{aligned} \partial _t\rho _i - \operatorname {div}\big (k_i\rho _i\nabla (\rho _1+\rho _2)\big ) = 0 \quad \text{ in } {\mathbb T}^d,\ t>0,\ i=1,2, \end{aligned}$$

where $\rho _i$ is the density of the *i*th population species, $k_i>0$ is a diffusion coefficient, ${\mathbb T}^d$ is the multidimensional torus, and we impose the initial conditions $\rho _i(0)=\rho _i^0$ in ${\mathbb T}^d$ for $i=1,2$. The equations have been suggested by Busenberg and Travis [7] to describe the segregation of populations, see also [4, 21]. They have also been proposed, based on interacting particle systems, to introduce short-range repulsion in cell-cell adhesion models [12, 33].

### Motivation and model setting

Our motivation for an approximation of (1) is to recover the entropy structure of (1) from the energy and entropy of the approximating fluid system. Indeed, it has been shown in [28, 29] that system (1) possesses *two* entropies, the Boltzmann–Shannon entropy $H_1$ and the Rao entropy $H_2$,

$$\begin{aligned} H_1 = \sum _{i=1}^2\int _{{\mathbb T}^d}k_i^{-1}\rho _i(\log \rho _i-1)dx, \quad H_2 = \frac{1}{2}\int _{{\mathbb T}^d}(\rho _1+\rho _2)^2 dx. \end{aligned}$$

This means that formally, along solutions to (1),

$$\begin{aligned} \frac{dH_1}{dt} + \int _{{\mathbb T}^d}|\nabla (\rho _1+\rho _2)|^2 dx&= 0, \\ \frac{dH_2}{dt} + \int _{{\mathbb T}^d}(k_1\rho _1+k_2\rho _2) |\nabla (\rho _1+\rho _2)|^2 dx&= 0. \end{aligned}$$

However, the origin of this double entropy structure remained unclear. We propose a fluiddynamical approximation that possesses the thermodynamical entropy $H_1$ and an energy containing $H_2$. Thus, the entropy structure of (1) originates from the energy and entropy of the associated fluiddynamical system.

Before we make this statement precise, we comment on the cross-diffusion system (1). The diffusion matrix associated to (1) has rank one, such that this system is of mixed hyperbolic–parabolic type. Indeed, we can reformulate (1) as a diffusion equation for the total density $\rho _1+\rho _2$ and a transport equation for one of the densities:

2 $$\begin{aligned}&\partial _t(\rho _1+\rho _2) = \operatorname {div}\big ((k_1\rho _1+k_2\rho _2)\nabla (\rho _1+\rho _2)\big ), \end{aligned}$$

3 $$\begin{aligned}&\partial _t\rho _i+\operatorname {div}(\rho _i\bar{u}_i)=0\quad \text{ in } {\mathbb T}^d,\ t>0,\ i=1,2, \end{aligned}$$

where $\bar{u}_i=-k_i\nabla (\rho _1+\rho _2)$ is the velocity associated to the *i*th species. Observe that the equations decouple if $k:=k_1=k_2$; then $\rho _1+\rho _2$ solves a porous-medium equation, and the densities $\rho _i$ are transported with the common velocity $\bar{u}=-k\nabla (\rho _1+\rho _2)$. We refer to [18, 19] for details on the hyperbolic–parabolic structure.

A fluiddynamical approximation of (1) takes the form

4 $$\begin{aligned} \begin{aligned}&\partial _t\rho _i + \operatorname {div}(\rho _i u_i) = 0, \quad i=1,2, \\&\varepsilon \partial _t(\rho _iu_i) + \varepsilon \operatorname {div}(\rho _iu_i\otimes u_i) = \varepsilon \operatorname {div}S - k_i^{-1}\rho _i u_i - \rho _i\nabla (\rho _1+\rho _2), \end{aligned} \end{aligned}$$

where $u_i$ is the partial velocity of the *i*th species, $\varepsilon >0$ is a small number, *S* is the stress tensor, $-k_i^{-1}\rho _iu_i$ is the relaxation term, and $-\rho _i\nabla (\rho _1+\rho _2)$ is a force term. The formal limit $\varepsilon \rightarrow 0$ in (4) leads to (2)–(3). As this limit is singular, its rigorous proof is delicate.

The main difficulty comes from the force term $-\rho _i\nabla (\rho _1+\rho _2)$, since the energy of the fluiddynamical equations does not provide any gradient estimate. This issue does not occur in the relaxation-time limit of the Euler–Poisson equations, since the force reads as $-\rho _i\nabla \Phi$, and the electric potential $\Phi$ solves the Poisson equation, thus providing sufficient regularity to apply the div–curl lemma [27]. The lack of a gradient bound can be overcome by allowing for a Korteweg term in (4), leading to Euler–Korteweg equations [32] or Navier–Stokes–Korteweg equations [8]. More precisely, we add to the right-hand side of (4) the expression

$$\begin{aligned} K = \varepsilon \rho _i\nabla \bigg (\kappa (\rho _i)\Delta \rho _i + \frac{1}{2}\kappa '(\rho _i)|\nabla \rho _i|^2\bigg ), \end{aligned}$$

where $\kappa (\rho _i)$ is the capillarity coefficient. In this paper, we choose $\kappa (\rho _i)=1/(2\rho _i)$, which leads to

$$\begin{aligned} K = \varepsilon \rho _i\nabla \bigg (\frac{\Delta \sqrt{\rho _i}}{\sqrt{\rho _i}}\bigg ). \end{aligned}$$

The expression $\Delta \sqrt{\rho _i}/\sqrt{\rho _i}$ is known in quantum mechanics as the Bohm potential, and equations (4) become the quantum Navier–Stokes equations studied in, e.g., [3, 24, 31, 35]. Other choices for $\kappa (\rho _i)$ are discussed in Remark 4. As in [24, 35], we use the density-dependent stress tensor $S=\rho _i\nabla u_i$. This dependence is needed in the derivation of the entropy inequality.

A second difficulty is due to the fact that we control the kinetic energy $\rho _i|u_i|^2$ in $L^1({\mathbb T}^d)$ only. This is not sufficient to prevent concentration phenomena in the convective term $\rho _i u_i\otimes u_i$. A way out is the introduction of additional drag forces like in [6, Sec. 9] and used in the context of the quantum Navier–Stokes equations in [35].

Summarizing, we perform the asymptotic limit $\varepsilon \rightarrow 0$ in the compressible Navier–Stokes equations with Korteweg regularization and drag forces:

5 $$\begin{aligned}&\partial _t\rho _i + \operatorname {div}(\rho _i u_i) = 0 \quad \text{ in } {\mathbb T}^d,\ t>0, \ i=1,2, \end{aligned}$$

6 $$\begin{aligned}&\varepsilon \partial _t(\rho _iu_i) + \varepsilon \operatorname {div}(\rho _iu_i\otimes u_i) = \varepsilon \rho _i\nabla \bigg ( \frac{\Delta \sqrt{\rho _i}}{\sqrt{\rho _i}}\bigg ) + \varepsilon \operatorname {div}(\rho _i\nabla u_i) \nonumber \\&\phantom {xx} -\varepsilon u_i - \varepsilon \rho _i u_i|u_i|^2 - k_i^{-1}\rho _iu_i - \rho _i\nabla (\rho _1+\rho _2), \end{aligned}$$

subject to the initial conditions

7 $$\begin{aligned} \rho _i(0) = \rho _i^0, \quad \rho _i(0)u_i(0) = \rho _i^0 u_i^0 \quad \text{ in } {\mathbb T}^d,\ i=1,2. \end{aligned}$$

In the following, we set $\rho =(\rho _1,\rho _2)$ and $u=(u_1,u_2)$.

### Key ideas

A priori estimates are derived by estimating the energy

8 $$\begin{aligned} E(\rho ,u) = \int _{{\mathbb T}^d}\bigg (\frac{1}{2}(\rho _1+\rho _2)^2 + \frac{\varepsilon }{2}\sum _{i=1}^2\rho _i|u_i|^2 + \varepsilon \sum _{i=1}^2|\nabla \sqrt{\rho _i}|^2\bigg )dx, \end{aligned}$$

which is the sum of the potential, kinetic, and Korteweg energies. A formal computation, made rigorous on the approximate level in Section 2.2, shows that

$$\begin{aligned} \frac{dE}{dt} + \sum _{i=1}^2\int _{{\mathbb T}^d}\big (k_i^{-1}\rho _i|u_i|^2 + \varepsilon \rho _i|\nabla u_i|^2 + \varepsilon |u_i|^2 + \varepsilon \rho _i|u_i|^4\big )dx = 0. \end{aligned}$$

Unfortunately, this equality does not provide any gradient bound for the densities independent of $\varepsilon$. Our main idea is to obtain such a bound from the entropy

9 $$\begin{aligned} H(\rho ) = \sum _{i=1}^2k_i^{-1}\int _{{\mathbb T}^d}\rho _i(\log \rho _i-1)dx. \end{aligned}$$

A formal computation (made rigorous for the approximate solutions in Section 2.3) shows that

$$\begin{aligned} \frac{dH}{dt} + \int _{{\mathbb T}^d}|\nabla (\rho _1+\rho _2)|^2 dx + \frac{\varepsilon }{2}\int _{{\mathbb T}^d}\rho _i|D^2\log \rho _i|^2 dx \le R, \end{aligned}$$

where the remainder *R* depends on the unknowns $\rho _i$, $u_i$ and their derivatives (like $\sqrt{\rho _i}\nabla u_i$), but it is independent of $\varepsilon$. The last integral on the left-hand side gives ($\varepsilon$-dependent) estimates in $H^2({\mathbb T}^d)$ and $W^{1,4}({\mathbb T}^d)$ from the inequality

10 $$\begin{aligned} \int _{{\mathbb T}^d}\rho _i|D^2\log \rho _i|^2 dx \ge c(d)\int _{{\mathbb T}^d}\big (|\Delta \sqrt{\rho _i}|^2 + |\nabla \root 4 \of {\rho _i}|^4\big ) dx \end{aligned}$$

for some $c(d)>0$, which holds for sufficiently smooth functions $\rho _i$; see [26, Lemma 2.2] and [24, Appendix] (or [35, Lemma 2.1]) for a proof. Then the remainder *R* can be controlled by the bounds coming from the energy and (10). Since the limiting system (1) may possess discontinuous solutions [5], we cannot expect gradient bounds for the individual densities $\rho _i$ but only for the sum $\rho _1+\rho _2$. Thus, we cannot expect better estimates.

Denoting by $(\rho ^\varepsilon ,u^\varepsilon )$ a weak solution to (5)–(7), the energy and entropy estimates together with the Aubin–Lions lemma yield strong convergence of the sum $\bar{\rho }^\varepsilon :=\rho _1^\varepsilon +\rho _2^\varepsilon$, but we have only weak convergence for $\rho _i^\varepsilon$ and $\nabla \bar{\rho }^\varepsilon$. Thus, the limit in the product $\rho _i^\varepsilon \nabla \bar{\rho }^\varepsilon$ cannot be easily identified.

We show two results. First, we prove that the strong limit $\bar{\rho }$ of $\bar{\rho }^\varepsilon$ solves (2) with a “defect”,

$$\begin{aligned} \partial _t\bar{\rho }- \operatorname {div}\big ((k_1\rho _1+k_2\rho _2)\nabla \bar{\rho }\big ) = (k_2-k_1)(k_2^{-1}J_2+\rho _2\nabla \bar{\rho }) \end{aligned}$$

where $\rho _i$ is the weak limit of $\rho _i^\varepsilon$ and $J_2$ is the weak limit of $\rho _2^\varepsilon u_2^\varepsilon$ (see Theorem 2). The right-hand side vanishes if $k_1=k_2$ or if we can identify $J_2$ with $-k_2\rho _2\nabla \bar{\rho }$. Unfortunately, we have not been able to prove this identification for general values of $k_1$, $k_2$. Indeed, neither the div–curl lemma nor Feireisl’s viscous flux approach can be applied because of the lack of suitable gradient bounds uniform in $\varepsilon$.

Second, we consider the case $k_1=k_2=1$. Then $\bar{\rho }$ solves the quadratic porous-medium equation (see (2)), but we still have no information about the evolution of $\rho _i$. To derive the dynamics, we apply the relative entropy method. The idea is to compare a solution $(\rho ^\varepsilon ,u^\varepsilon )$ to (5)–(6) with a solution $(\bar{\rho },\bar{u})$ to the limit system (2)–(3) with $k_1=k_2=1$ and $\bar{u}=-\nabla \bar{\rho }$. The relative entropy (more precisely: relative energy) is defined by

$$\begin{aligned} E_R(\rho ^\varepsilon ,u^\varepsilon |\bar{\rho },\bar{u}) = \int _{{\mathbb T}^d}\bigg \{\frac{1}{2}(\bar{\rho }^\varepsilon -\bar{\rho })^2 + \varepsilon \sum _{i=1}^2\bigg (\frac{\rho _i^\varepsilon }{2}|u_i^\varepsilon -\bar{u}|^2 + |\nabla (\rho _i^\varepsilon )^{1/2}|^2\bigg )\bigg \}dx. \end{aligned}$$

A computation, made rigorous in Section 4, shows that

$$\begin{aligned} \frac{dE_R}{dt}(\rho ^\varepsilon ,u^\varepsilon |\bar{\rho },\bar{u}) + \sum _{i=1}^2\int _{{\mathbb T}^d}\rho _i^\varepsilon |u_i^\varepsilon -\bar{u}|^2 dx \le C\root 4 \of {\varepsilon }. \end{aligned}$$

Here, we need the assumption $k_1=k_2$. We infer that $(\rho _i^\varepsilon )^{1/2}(u_i^\varepsilon -\bar{u})\rightarrow 0$ strongly in $L^2(0,T;L^2({\mathbb T}^d))$, which implies that $\rho _i^\varepsilon u_i^\varepsilon \rightharpoonup \rho _i\bar{u}$ weakly in $L^2(0,T;L^{4/3}({\mathbb T}^d))$. This allows us to identify $J_i$ with $\rho _i\bar{u}=-\rho _i\nabla \bar{\rho }$, and $\rho _i$ solves the transport equation (3).

### State of the art

There are many results in the literature on the relaxation limit in hyperbolic systems. General results can be found, for instance, in [17]. The relaxation limit in Euler–Poisson systems, leading to the drift-diffusion equations, was proved in [27], exploiting the regularizing effect of the Poisson equation. Relaxation-time limits were also performed in Euler–Maxwell [34] and quantum hydrodynamic equations [25]. Using compactness methods, relaxation limits in the compressible Navier–Stokes–Poisson equations [30] and in the quantum Navier–Stokes equations [3] were proved. Note that our limit is more delicate since the regularizing terms vanish in the limit.

The relative entropy method was first used by Dafermos [15] and Di Perna [16]. It was extended later by Lattanzio and Tzavaras [32] to compare the solution to the frictional Euler equations with the solution to the porous-medium equation. This technique was also applied in the analysis of the high-friction regime of Euler–Korteweg equations [23], for more general aggregation-diffusion equations [13], compressible Navier–Stokes–Korteweg systems [8], and Euler–Riesz models [1, 2].

The Busenberg–Travis system (1) can be derived from interacting particle systems in the mean-field limit, even for an arbitrary number of species [14] and for nonlinear pressures [10]. In the general case, the limiting system reads as

11 $$\begin{aligned} \partial _t u_i = \operatorname {div}(u_i\nabla p_i(u)), \quad p_i(u) = \sum _{j=1}^n a_{ij}u_j\quad \text{ in } {\mathbb T}^d,\ i=1,\ldots ,n, \end{aligned}$$

where $a_{ij}\ge 0$ are some numbers. If the matrix $(a_{ij})$ is positive definite, a global existence analysis can be found in [28]. If the matrix $(a_{ij})$ is of rank one, i.e. $a_{ij}=k_i$ like in our situation, the existence of global classical solutions to (1) (under the positivity assumption $\sum _{i=1}^n\rho _i^0\ge c>0$) was proved in [19]. The positivity ensures the regularity of the total density. If the positivity assumption is relaxed to nonnegativity, the existence of global measure-valued solutions can be shown [22]. Steady states may be discontinuous [11], and there is some numerical evidence [5] that this may be also true for transient solutions. The lack of regularity motivated the authors of [9] to work in the one-dimensional setting with solutions of bounded variation.

The limit in (5)–(6) seems to be new, and we believe that it contributes to the understanding of the entropy structure of the Busenberg–Travis cross-diffusion system and possibly of related models.

### Main results

We first define our notion of weak solution. This is necessary since the Korteweg term in (6) needs special care.

#### Definition 1

We call $(\rho ,u)$ with $\rho =(\rho _1,\rho _2)$ and $u=(u_1,u_2)$ a *weak solution* to (5)–(7) on (0, *T*) if, for $i=1,2$, $\rho _i\ge 0$ in ${\mathbb T}^d\times (0,T)$,

$$\begin{aligned}&\rho _i\in L^\infty (0,T;L^2({\mathbb T}^d)), \quad \sqrt{\rho _i}u_i\in L^\infty (0,T;L^2({\mathbb T}^d)), \\&\sqrt{\rho _i}\in L^2(0,T;H^2({\mathbb T}^d)), \quad \rho _1+\rho _2\in L^2(0,T;H^1({\mathbb T}^d)), \\&\sqrt{\rho _i}|\nabla u_i|\in L^2(0,T;L^2({\mathbb T}^d)), \quad \root 4 \of {\rho _i}|u_i|\in L^4(0,T;L^4({\mathbb T}^d)), \end{aligned}$$

for all $\phi \in C_0^\infty ({\mathbb T}^d\times [0,T))$ and $\psi \in C_0^\infty ({\mathbb T}^d\times [0,T);{\mathbb R}^d)$,

12 $$\begin{aligned} 0&= -\int _0^T\int _{{\mathbb T}^d}\rho _i\partial _t\phi dxdt - \int _{{\mathbb T}^d}\rho _i^0\phi (0)dx - \int _0^T\int _{{\mathbb T}^d}\rho _i u_i\cdot \nabla \phi dxdt, \end{aligned}$$

13 $$\begin{aligned} 0&= -\varepsilon \int _0^T\int _{{\mathbb T}^d}\rho _i u_i\cdot \partial _t\psi dxdt - \varepsilon \int _{{\mathbb T}^d}\rho _i^0 u_i^0\cdot \psi (0)dx - \varepsilon \int _0^T\int _{{\mathbb T}^d}\rho _i(u_i\otimes u_i):\nabla \psi dxdt \nonumber \\&\phantom {xx}+ \varepsilon \int _0^T\int _{{\mathbb T}^d}\rho _i\nabla u_i:\nabla \psi dxdt + \varepsilon \int _0^T\int _{{\mathbb T}^d}\Delta \sqrt{\rho _i}\big (2\nabla \sqrt{\rho _i} \cdot \psi + \sqrt{\rho _i}\operatorname {div}\psi \big )dxdt \nonumber \\&\phantom {xx} + \varepsilon \int _0^T\int _{{\mathbb T}^d}(u_i + \rho _i|u_i|^2u_i)\cdot \psi dxdt + \int _0^T\int _{{\mathbb T}^d} \big (k_i^{-1}\rho _i u_i + \rho _i\nabla (\rho _1+\rho _2)\big ) \cdot \psi dxdt, \end{aligned}$$

and the initial conditions (7) hold in the sense of distributions.

We impose the following assumptions:

1. Parameter: $$d=1,2,3$$, $$T>0$$, and $$k_1>0$$, $$k_2>0$$.
2. Initial data: $\rho _i^0\in L^2({\mathbb T}^d)$, $\sqrt{\rho _i^0}|u_i^0|\in L^2({\mathbb T}^d)$, $\sqrt{\rho _i^0}\in H^1({\mathbb T}^d)$, $\log \rho _i^0\in L^1({\mathbb T}^d)$ for $i=1,2$.

The assumption of at most $d=3$ space dimensions is due to Sobolev embeddings (we need $H^2({\mathbb T}^d)\hookrightarrow L^\infty ({\mathbb T}^d)$ in Lemma 10). Our analysis strongly depends on inequality (10). To avoid boundary integrals, we consider equations (5)–(6) on the torus. The regularity of the initial data is needed to obtain a finite initial energy and entropy. We may allow for reaction terms in (5) if the reactions depend nonlinearly on the total density $\rho _1+\rho _2$ only (since we have strong convergence only for the sum). We discuss further generalizations of the nonlinearities in Remark 4.

Our first main result reads as follows.

#### Theorem 1

(Existence of solutions). Let Assumptions (A1)–(A2) hold. Then there exists a weak solution $(\rho ,u)$ to (5)–(7) with $\rho =(\rho _1,\rho _2)$ and $u=(u_1,u_2)$ such that

14 $$\begin{aligned} E(\rho (t),u(t))&+ \sum _{i=1}^2\int _0^t\int _{{\mathbb T}^d} k_i^{-1}\rho _i|u_i|^2 dxds \end{aligned}$$

15 $$\begin{aligned}&+ \varepsilon \sum _{i=1}^2\int _0^t\int _{{\mathbb T}^d} \big (\rho _i|\nabla u_i|^2 + |u_i|^2 + \rho _i|u_i|^4\big )dxds \le E(\rho ^0,u^0), \nonumber \\{H(\rho (t))}&+ C_1(d)\varepsilon \int _0^t\int _{{\mathbb T}^d}\big (|\Delta \sqrt{\rho _i}|^2 + |\nabla \root 4 \of {\rho _i}|^4\big )dxds \le {C_2(\rho ^0,u^0)}, \end{aligned}$$

where $C_1(d)>0$ is a constant only depending on the space dimension *d*, $C_2(\rho ^0,u^0)>0$ depends on the initial data (but not on $\varepsilon$), and we recall definitions (8) and (9) of *E* and *H*. Moreover, we have the regularity

$$\begin{aligned} \partial _t\rho _i\in L^2(0,T;L^2({\mathbb T}^d)), \quad \partial _t(\rho _iu_i)\in L^{4/3}(0,T;H^s({\mathbb T}^d)'), \quad s>d/2+1. \end{aligned}$$

Therefore, the initial condition for $\rho _i$ holds a.e. in ${\mathbb T}^d$, and the initial condition for $\rho _iu_i$ holds in $H^s({\mathbb T}^d)'$.

The proof of the existence of solutions is based on an approximate scheme. We add the parabolic regularization $\delta \Delta \rho _i$ to the mass balance equation (5) and the regularization $\delta \varepsilon \operatorname {div}(u_i\otimes \nabla \rho _i)$ to the momentum balance equation (similarly as in the existence analysis for the compressible Navier–Stokes equations; see [20, Sec. 7.2]). The latter term is needed to compensate some contributions coming from the parabolic regularization when deriving the energy inequality. The local existence of solutions is shown by the Faedo–Galerkin method. The solution can be extended to a global one thanks to the energy estimate. The entropy production in (15) is obtained by applying inequality (10).

If $k_1=k_2$, a computation similar to the proof of Lemma 8 shows that the entropy inequality (15) can be improved by replacing $C_2(\rho ^0,u^0)$ by $H(\rho ^0)+C\root 4 \of {\varepsilon }$, where $C>0$ is independent of $\varepsilon$.

#### Theorem 2

(Limit $\varepsilon \rightarrow 0$). Let $(\rho ^\varepsilon ,u^\varepsilon )$ be a weak solution to (5)–(7) as constructed in Theorem 1. Then there exists a subsequence (not relabeled) such that for $i=1,2$,

$$\begin{aligned} \rho _i^\varepsilon \rightharpoonup \rho _i&\quad \text{ weakly* } \text{ in } L^\infty (0,T;L^2({\mathbb T}^d)), \\ \rho _1^\varepsilon +\rho _2^\varepsilon \rightarrow \rho _1+\rho _2&\quad \text{ strongly } \text{ in } L^2(0,T;L^2({\mathbb T}^d)), \\ \rho _i^\varepsilon u_i^\varepsilon \rightharpoonup J_i&\quad \text{ weakly } \text{ in } L^2(0,T;L^{4/3}({\mathbb T}^d)), \end{aligned}$$

and $$\bar{\rho }:=\rho _1+\rho _2$$ solves

$$\begin{aligned} \partial _t\bar{\rho }- \operatorname {div}\big ((k_1\rho _1+k_2\rho _2)\nabla \bar{\rho })\big ) = -(k_2-k_1)\operatorname {div}(k_2^{-1}J_2+\rho _2\nabla \bar{\rho }) \quad \text{ in } {\mathbb T}^d,\ t>0, \end{aligned}$$

with the initial condition $\bar{\rho }(0)=\rho _1^0+\rho _2^0$ in the sense of $W^{1,4}({\mathbb T}^d)'$.

The proof is based on the energy and entropy estimates proved in Theorem 1 and on compactness arguments. If $k_1=k_2$, we can prove a stronger result, using the relative entropy method. Indeed, as explained in Section 1.2, we are able to prove that not only $\rho _i^\varepsilon u_i^\varepsilon \rightharpoonup J_i$ weakly in $L^2(0,T;L^{4/3}({\mathbb T}^d))$ (which follows from the energy inequality) but also $\rho _i^\varepsilon u_i^\varepsilon \rightharpoonup \rho _i\bar{u}$ weakly in $L^1(0,T;L^1({\mathbb T}^d))$ (which follows from the relative entropy inequality). This allows us to identify $J_i=\rho _i\bar{u}=-\rho _i\nabla \bar{\rho }$.

#### Theorem 3

(Limit $\varepsilon \rightarrow 0$, $k_1=k_2$). Let $k_1=k_2=1$, let $(\rho ^\varepsilon ,u^\varepsilon )$ be a weak solution to (5)–(7) as constructed in Theorem 1, and let $(\bar{\rho },\bar{u})$ be the unique smooth solution to

$$\begin{aligned} \partial _t\bar{\rho }+ \operatorname {div}(\bar{\rho }\bar{u}) = 0, \quad \bar{u} = -\nabla \bar{\rho }\quad \text{ in } {\mathbb T}^d,\ t>0, \end{aligned}$$

with initial conditions $\bar{\rho }(0)=\rho _1^0+\rho _2^0$ (this requires smooth positive initial data). Then, as $\varepsilon \rightarrow 0$,

$$\begin{aligned} \rho _1^\varepsilon +\rho _2^\varepsilon \rightarrow \bar{\rho }&\quad \text{ strongly } \text{ in } L^2(0,T;L^2({\mathbb T}^d)), \\ \rho _i^\varepsilon u_i^\varepsilon \rightharpoonup \rho _i\bar{u}&\quad \text{ weakly } \text{ in } L^2(0,T;L^{4/3}({\mathbb T}^d)). \end{aligned}$$

and the $L^2({\mathbb T}^d)$-weak limit $\rho _i$ of $(\rho _i^\varepsilon )$ solves the transport equation

$$\begin{aligned} \partial _t\rho _i - \operatorname {div}(\rho _i\nabla (\rho _1+\rho _2)) = 0 \quad \text{ in } {\mathbb T}^d,\ t>0,\ i=1,2. \end{aligned}$$

#### Remark 4

*(Generalizations).* Our results can be extended in various directions. First, all results are valid for more than two species, and it is sufficient to replace the sum over $i=1,2$ by $i=1,\ldots ,n$, where $n\in {\mathbb N}$ is arbitrary. Second, one may try more general Korteweg functions $\kappa (\rho _i)$. A simple choice is $\kappa (\rho _i)=1$, giving the higher-order regularization $K=\varepsilon \rho _i\nabla \Delta \rho _i$, which equals the flux of the thin-film equation. The Korteweg energy density becomes $\varepsilon |\nabla \rho _i|^2$ and the entropy production simplifies to $\varepsilon \int _{{\mathbb T}^d}(\Delta \rho _i)^2 dx$, thus providing $H^2({\mathbb T}^d)$ bounds for $\rho _i$. Since we do not need inequalities like (10) in this case, we may allow for bounded domains instead of the torus. However, one needs to check whether this regularization is sufficient to pass to the limit $\varepsilon \rightarrow 0$ in the regularizing terms, as such a choice does not provide gradient bounds for $\sqrt{\rho _i}$ or $\root 4 \of {\rho _i}$. We leave this question to future works. Third, we can derive the generalized Busenberg–Travis system (11) if the matrix $(a_{ij})$ is symmetric and positive definite. This system is fully parabolic, which simplifies the asymptotic analysis $\varepsilon \rightarrow 0$. Indeed, we set in (6) $k_i=1$ and replace the term $-\rho _i\nabla (\rho _1+\rho _2)$ by $\rho _i\nabla p_i(u)$, where $p_i(u)$ is defined in (11). Then, using the test function $\nabla \log \rho _i$ in the weak formulation of (6) and summing over $i=1,\ldots ,n$ to derive the entropy inequality, we find that

$$\begin{aligned} -\sum _{i=1}^n\int _{{\mathbb T}^d}\rho _i\nabla p_i(u)\cdot \nabla \log \rho _i dx = -\sum _{i,j=1}^n\int _{{\mathbb T}^d}a_{ij}\nabla \rho _i\cdot \nabla \rho _j dx \le -\alpha \sum _{i=1}^n\int _{{\mathbb T}^d}|\nabla \rho _i|^2 dx, \end{aligned}$$

where $\alpha >0$ is the smallest eigenvalue of $(a_{ij})$. This provides uniform gradient bounds for each $\rho _i$, and the Aubin–Lions compactness lemma implies the strong convergence of the approximating sequence of $\rho _i$, thus allowing us to perform the limit $\varepsilon \rightarrow 0$. $\square$

The paper is organized as follows. We prove the existence of global weak solutions (Theorem 1) in Section 2. The limit $\varepsilon \rightarrow 0$ for general $k_1$, $k_2>0$ (Theorem 2) is shown in Section 3, while Section 4 is devoted to the proof of Theorem 3 in the special case $k_1=k_2$.

## Proof of Theorem 1: existence of solutions

We show first the existence of solutions locally in time and then derive uniform estimates from the energy (8) and entropy (9), which allows us to extend the local solutions globally.

### Local existence of solutions

The local-in-time existence of solutions can be proven by the Faedo–Galerkin method; see [20, Chap. 7] for the compressible Navier–Stokes equations and [24] for the quantum Navier–Stokes equations. Since the proof is very similar to these works, we only sketch it.

We regularize the initial data by taking $(\rho ^0,u^0)\in C^\infty ({\mathbb T}^d;{\mathbb R}^4)$ such that $\rho _i^0\ge c>0$ for some $c>0$ ($i=1,2$). This is possible by using some mollifier with parameter $\delta >0$, proving the result for this initial datum and then passing to the limit $\delta \rightarrow 0$. Let $T>0$, let $(e_k)$ be an orthonormal basis of $L^2({\mathbb T}^d)$ which is also an orthogonal basis of $H^1({\mathbb T}^d)$, and set $X_N=\operatorname {span}\{e_1,\ldots ,e_N\}$. Let the velocity $u=(u_1,u_2)\in C^0([0,T];X_N^2)$ be given and solve the approximate equations

16 $$\begin{aligned} \partial _t \rho ^N_i + \operatorname {div}(\rho ^N_i u_i) = \delta \Delta \rho ^N_i, \quad \rho ^N_i(0)=\rho _i^0\quad \text{ in } {\mathbb T}^d\times (0,T),\ i=1,2. \end{aligned}$$

The maximum principle provides the lower and upper bounds $0<r_i\le \rho _i^N\le R_i$ in ${\mathbb T}^d\times (0,T)$, $i=1,2$, where $r_i$ and $R_i$ depend on $\delta$ and the $L^\infty ({\mathbb T}^d)$ norm of $\operatorname {div}u_i$, and $r_i$ additionally depends on the lower bound $c>0$ of the initial data. We introduce the operator $S:C^0([0,T];X_N^2)\rightarrow C^0([0,T];C^3({\mathbb T}^d;{\mathbb R}^2))$ by $S(u)=\rho ^N=(\rho ^N_1,\rho ^N_2)$. This operator is Lipschitz continuous.

Next, we solve the momentum equation on the space $X_N^2$. We are looking for a solution $u^N=(u_1^N,u_2^N)\in C^0([0,T];X_N^2)$ such that for any $\psi \in C^1([0,T];X_N^2)$ with $\psi (T)=0$ and $i=1,2$,

17 $$\begin{aligned} -\varepsilon&\int _{{\mathbb T}^d}\rho _i^0 u_i^0\cdot \psi (0)dx = \varepsilon \int _0^T\int _{{\mathbb T}^d}\bigg \{\rho ^N_i u_i^N\cdot \partial _t\psi + \rho _i^N(u_i^N\otimes u_i^N):\nabla \psi \nonumber \\&+ \rho _i^N\nabla \bigg (\frac{\Delta (\rho _i^N)^{1/2}}{(\rho _i^N)^{1/2}} \bigg )\cdot \psi - \rho _i^N\nabla u_i^N:\nabla \psi - u_i^N\cdot \psi - \rho _i^N|u_i^N|^2u_i^N\cdot \psi \bigg \}dxdt \nonumber \\&- \int _0^T\int _{{\mathbb T}^d}\bigg (\frac{1}{k_i}\rho _i^Nu_i^N + \rho _i^N\nabla (\rho _1^N+\rho _2^N)\bigg )\cdot \psi dxdt - \delta \varepsilon \int _0^T\int _{{\mathbb T}^d}(u_i^N\otimes \nabla \rho _i^N):\nabla \psi dxdt. \end{aligned}$$

As already mentioned in the introduction, the additional term $\delta \varepsilon \operatorname {div}(u_i^N\otimes \nabla \rho _i^N)$ is introduced to deal with the term $\delta \Delta \rho _i^N$ in (16) when deriving the energy estimates; see the proof of Lemma 6. To solve problem (17), we introduce the operator family

$$\begin{aligned} \mathcal {M}[\eta ]:X_N\rightarrow X_N', \quad \langle \mathcal {M}[\eta ]u,w\rangle = \int _{{\mathbb T}^d}\eta u\cdot w dx, \end{aligned}$$

where $\eta \in L^1({\mathbb T}^d)$ satisfies $\eta \ge r:=\min \{r_1,r_2\}>0$ and $u,w\in X_N$. The operator $\mathcal {M}$ is invertible and $\mathcal {M}^{-1}$ is Lipschitz continuous as a function from $L^1({\mathbb T}^d)$ to the space of bounded linear mappings $X_N'\rightarrow X_N$.

We can rephrase the integral equation (17) as an ordinary differential equation on $X_N$,

18 $$\begin{aligned} \frac{d}{dt}\big (\mathcal {M}[\rho ^N_i]u^N_i\big ) = \mathcal {N}[u,u^N], \quad \mathcal {M}[\rho ^0_i]u_i^N(0) = \mathcal {M}[\rho _i^0]u_i^0, \end{aligned}$$

where $$\rho ^N=S(u)$$ and

$$\begin{aligned} \mathcal {N}[u,u^N]&= -\operatorname {div}(\rho ^N_i u\otimes u_i^N) + \operatorname {div}(\rho _i^N\nabla u_i^N) + \rho _i^N\nabla \bigg (\frac{\Delta (\rho _i^N)^{1/2}}{(\rho _i^N)^{1/2}} \bigg ) \\&\phantom {xx}- u_i^N - \rho _i^N|u_i|^2u_i^N - \varepsilon ^{-1}\big (k_i^{-1}\rho _i^Nu_i^N + \rho _i^N\nabla (\rho _1^N+\rho _2^N)\big ) + \delta \operatorname {div}(u_i^N\otimes \nabla \rho _i^N). \end{aligned}$$

By standard theory for systems of ordinary differential equations, for given $$u\in C^0([0,T];$$ $$X_N^2)$$, there exists a unique solution $$u^N\in C^1([0,T];X_N^2)$$. We are looking for a fixed point $$u=u^N$$ of (18), and the fixed-point equation can be written in integrated form as

$$\begin{aligned} u_i^N(t) = \mathcal {M}^{-1}[S(u^N)_i(t)] \bigg (\mathcal {M}[\rho _i^0]u_i^0 + \int _0^t\mathcal {N}[u^N,u^N]ds\bigg )\quad \text{ in } X_N \end{aligned}$$

and using the Lipschitz continuity of *S*, $\mathcal {M}^{-1}$, and $\mathcal {N}$, we can apply Banach’s fixed-point theorem on a short time interval $[0,T^*]$ for some $0<T^*\le T$ in the space $C^0([0,T^*];X_N^2)$.

Before, we proceed with the uniform estimates, we recall the following classical lemma, which is used several times in this work.

#### Lemma 5

Let *v* be a weak solution to $\partial _t v + \operatorname {div}F = g$ in ${\mathbb T}^d$, $t>0$, for some integrable functions *F* and *g* in the sense of

$$\begin{aligned} 0 = -\int _0^T\int _{{\mathbb T}^d}v\partial _t\chi dxdt - \int _{{\mathbb T}^d}v(0)\chi (0)dx - \int _0^T\int _{{\mathbb T}^d}(F\cdot \nabla \chi + g\chi )dxdt \end{aligned}$$

for functions $\chi \in C_0^\infty ({\mathbb T}^d\times [0,T))$. Then, for any $t\in [0,T]$ and $\phi \in C^\infty ({\mathbb T}^d\times [0,T])$,

$$\begin{aligned} 0 = -\int _0^t\int _{{\mathbb T}^d}v\partial _s\phi dxds + \int _{{\mathbb T}^d}v\phi \Big |^{s=t}_{s=0}dx - \int _0^t\int _{{\mathbb T}^d}(F\cdot \nabla \phi + g\phi )dxds. \end{aligned}$$

### Approximate energy inequality

To prove the global existence of solutions, it is sufficient to show that the sequence $(u^N(t))$ is bounded in $X_N^2$ for $t\in [0,T^*]$ uniformly in $T^*$.

#### Lemma 6

(Energy inequality). Let $(\rho ^N,u^N)$ be the local solution to (16)–(17) constructed in Section 2.1. Then

$$\begin{aligned} \frac{dE}{dt}&(\rho ^N,u^N) + \sum _{i=1}^2k_i^{-1}\int _{{\mathbb T}^d}\rho _i^N|u_i^N|^2 dx + \varepsilon \sum _{i=1}^2\int _{{\mathbb T}^d}\big (|u_i^N|^2 + \rho _i^N|\nabla u_i^N|^2 + \rho _i^N|u_i^N|^4\big )dx \\&+ \delta \int _{{\mathbb T}^d}| \nabla (\rho _1^N+\rho _2^N)|^2dx + \frac{\delta \varepsilon }{2}\sum _{i=1}^2\int _{{\mathbb T}^d} \rho _i^N|D^2\log \rho _i^N|^2 dx = 0, \end{aligned}$$

recalling Definition (8) of $E(\rho ^N,u^N)$.

Since the lower bound $\rho _i^N\ge r_i>0$ yields a uniform estimate for $(u_i^N)$ in $L^2({\mathbb T}^d)$ uniformly in time, thanks to the definition of the energy (8), we obtain the desired estimate for $u^N$.

#### Proof

We use the test function $\phi = \varepsilon |u_i^N|^2/2-\varepsilon \Delta (\rho _i^N)^{1/2}/(\rho _i^N)^{1/2}+(\rho _1^N+\rho _2^N)$ in the weak formulation of (16) and the test function $\psi =u_i^N$ in the time-differentiated form of (17) (see Lemma 5) and add both equations. Then, using the identity $\partial _t(\rho _i^N u_i^N)+\operatorname {div}(\rho _i^N u_i^N\otimes u_i^N) = \rho _i^N(\partial _t u_i^N+u_i^N\cdot \nabla u_i^N) + \delta \Delta \rho _i^N u_i^N$ and proceeding as in the proof of [24, Lemma 3.1], some terms cancel, and we end up with

$$\begin{aligned} \varepsilon \frac{d}{dt}&\int _{{\mathbb T}^d}\big (\rho _i^N|u_i^N|^2 + |\nabla (\rho _i^N)^{1/2}|^2\big )dx + \int _{{\mathbb T}^d}\partial _t\rho _i^N(\rho _1^N+\rho _2^N)dx \\&= -\int _{{\mathbb T}^d}\big (k_i^{-1}\rho _i^N|u_i^N|^2 + \varepsilon (|u_i^N|^2 + \rho _i^N|\nabla u_i^N|^2 + \rho _i^N|u_i^N|^4)\big )dx \\&\phantom {xx}- \delta \varepsilon \int _{{\mathbb T}^d}\Delta \rho _i^N \frac{\Delta (\rho _i^N)^{1/2}}{(\rho _i^N)^{1/2}}dx - \delta \int _{{\mathbb T}^d}\nabla \rho _i^N\cdot \nabla (\rho _1^N+\rho _2^N)dx. \end{aligned}$$

Adding these equations for $i=1,2$ and using

19 $$\begin{aligned} \int _{{\mathbb T}^d}\Delta \rho _i^N\frac{\Delta (\rho _i^N)^{1/2}}{(\rho _i^N)^{1/2}} dx = \frac{1}{2}\int _{{\mathbb T}^d}\rho _i^N|D^2\log \rho _i^N|^2 dx \end{aligned}$$

(see [24, (3.7)]), we find that

$$\begin{aligned} \frac{d}{dt}&\int _{{\mathbb T}^d}\bigg (\varepsilon \sum _{i=1}^2\big (\rho _i^N|u_i^N|^2 + |\nabla (\rho _i^N)^{1/2}|^2\big ) + \frac{1}{2}(\rho _1+\rho _2)^2\bigg )dx \\&+ \sum _{i=1}^2\int _{{\mathbb T}^d}\big (k_i^{-1}\rho _i^N|u_i^N|^2 + \varepsilon (|u_i^N|^2 + \rho _i^N|\nabla u_i^N|^2 + \rho _i^N|u_i^N|^4)\big )dx \\&+ \delta \int _{{\mathbb T}^d}|\nabla (\rho _1^N+\rho _2^N)|^2dx + \frac{\delta \varepsilon }{2}\sum _{i=1}^2\int _{{\mathbb T}^d}\rho _i^N |D^2\log \rho _i^N|^2 dx = 0. \end{aligned}$$

This finishes the proof. $\square$

The energy inequality and inequality (10) imply the following bounds.

#### Corollary 7

(Uniform estimates I). Let $(\rho ^N,u^N)$ be the local solution to (16)–(17) constructed in Section 2.1. Then there exists $C>0$ independent of $(\delta ,\varepsilon ,N)$ such that for $i=1,2$,

$$\begin{aligned} \Vert \rho _i^N\Vert _{L^\infty (0,\infty ;L^2({\mathbb T}^d))} + \Vert (\rho _i^N)^{1/2}u_i^N\Vert _{L^2(0,\infty ;L^2({\mathbb T}^d))} + \sqrt{\varepsilon }\Vert \nabla (\rho _i^N)^{1/2}\Vert _{L^\infty (0,\infty ;L^2({\mathbb T}^d))}&\le C, \\ \sqrt{\varepsilon }\Vert (\rho _i^N)^{1/2}u_i^N\Vert _{L^\infty (0,\infty ;L^2({\mathbb T}^d))} + \sqrt{\varepsilon }\Vert (\rho _i^N)^{1/2}\nabla u_i^N\Vert _{L^2(0,\infty ;L^2({\mathbb T}^d))}&\le C, \\ \sqrt{\varepsilon }\Vert u_i^N\Vert _{L^2(0,\infty ;L^2({\mathbb T}^d))} + \root 4 \of {\varepsilon }\Vert (\rho _i^N)^{1/4}u_i^N\Vert _{L^4(0,\infty ;L^4({\mathbb T}^d))}&\le C, \\ \sqrt{\delta \varepsilon }\Vert (\rho _i^N)^{1/2}D^2\log \rho _i^N\Vert _{L^2(0,\infty ; L^2({\mathbb T}^d))} + \root 4 \of {\delta \varepsilon } \Vert \nabla (\rho _i^N)^{1/4}\Vert _{L^4(0,\infty ;L^4({\mathbb T}^d))}&\le C. \end{aligned}$$

### Approximate entropy inequality

Further uniform estimates are derived from the entropy inequality. Here, we work directly with the weak formulation of (16) and with the weak formulation (17).

#### Lemma 8

(Entropy inequality). Let $(\rho ^N,u^N)$ be the local solution to (16)–(17) constructed in Section 2.1. Then

$$\begin{aligned} H(\rho ^N&(t)) + \int _0^t\int _{{\mathbb T}^d} |\nabla (\rho _1^N+\rho _2^N)|^2 dxds + \frac{\varepsilon }{8}\sum _{i=1}^2\int _0^t\int _{{\mathbb T}^d} \rho _i^N|D^2\log \rho _i^N|^2 dxds \\&+ \sum _{i=1}^2\frac{4\delta }{k_i}\int _0^t\int _{{\mathbb T}^d} |\nabla (\rho _i^N)^{1/2}|^2dxds + \delta \varepsilon \sum _{i=1}^2\int _0^t\int _{{\mathbb T}^d}|\nabla \log \rho _i^N|^2dxds \le C, \end{aligned}$$

and the constant $C>0$ only depends on the initial data.

#### Proof

Recalling that $\rho _i^N$ is smooth and positive, we compute

20 $$\begin{aligned} H(\rho ^N&(t)) - H(\rho ^N(0)) = \sum _{i=1}^2\frac{1}{k_i}\int _0^t\int _{{\mathbb T}^d}\partial _s\rho _i^N \log \rho _i^N dxds \nonumber \\&= \sum _{i=1}^2\frac{1}{k_i}\int _0^t\int _{{\mathbb T}^d}\big (\rho _i^N u_i^N - \delta \nabla \rho _i^N\big )\cdot \nabla \log \rho _i^N dxds \nonumber \\&= \sum _{i=1}^2\frac{1}{k_i}\int _0^t\int _{{\mathbb T}^d}u_i^N \cdot \nabla \rho _i^N dxds - \sum _{i=1}^2\frac{4\delta }{k_i}\int _0^t\int _{{\mathbb T}^d} |\nabla (\rho _i^N)^{1/2}|^2 dxds. \end{aligned}$$

To estimate the first term on the right-hand side, we use the test function $\nabla \log \rho _i^N$ in (17):

21 $$\begin{aligned}&\frac{1}{k_i}\int _0^t\int _{{\mathbb T}^d}u_i^N\cdot \nabla \rho _i^N dxds = \frac{1}{k_i}\int _0^t\int _{{\mathbb T}^d}(\rho _i^Nu_i^N) \cdot \nabla \log \rho _i^N dxds = I_1+\cdots +I_7, \nonumber \\&I_1 = -\varepsilon \int _0^t\int _{{\mathbb T}^d}\big (\partial _s(\rho _i^N u_i^N) + \operatorname {div}(\rho _i^N u_i^N\otimes u_i^N)\big )\cdot \nabla \log \rho _i^N dxds, \nonumber \\&I_2 = \varepsilon \int _0^t\int _{{\mathbb T}^d}\rho _i^N\nabla \frac{\Delta (\rho _i^N)^{1/2}}{(\rho _i^N)^{1/2}} \cdot \nabla \log \rho _i^N dxds, \nonumber \\&I_3 = \varepsilon \int _0^t\int _{{\mathbb T}^d}\operatorname {div}(\rho _i^N\nabla u_i^N) \cdot \nabla \log \rho _i^N dxds, \nonumber \\&I_4 = -\varepsilon \int _0^t\int _{{\mathbb T}^d}u_i^N\cdot \nabla \log \rho _i^N dxds, \nonumber \\&I_5 = -\varepsilon \int _0^t\int _{{\mathbb T}^d}\rho _i^N|u_i^N|^2u_i^N \cdot \nabla \log \rho _i^N dxds, \nonumber \\&I_6 = -\int _0^t\int _{{\mathbb T}^d}\rho _i^N\nabla (\rho _1^N+\rho _2^N) \cdot \nabla \log \rho _i^N dxds, \nonumber \\&I_7 = \delta \varepsilon \int _0^t\int _{{\mathbb T}^d}\operatorname {div}(u_i^N\otimes \nabla \rho _i^N) \cdot \nabla \log \rho _i^N dxds. \end{aligned}$$

*Step 1: Estimation of* $I_1$, $I_2$, *and* $I_7$: We start with $I_2$. We infer from identity (19) that

$$\begin{aligned} I_2 = -\varepsilon \int _0^t\int _{{\mathbb T}^d}\frac{\Delta (\rho _i^N)^{1/2}}{ (\rho _i^N)^{1/2}}\Delta \rho _i^N dxds = -\frac{\varepsilon }{2}\int _0^t\int _{{\mathbb T}^d}\rho _i^N|D^2\log \rho _i^N|^2 dxds, \end{aligned}$$

and this expression will be used to absorb some integrals coming from the other terms. It follows from (16) that

22 $$\begin{aligned} I_1&= -\varepsilon \int _0^t\int _{{\mathbb T}^d}\big (\rho _i^N(\partial _s u_i^N + u_i^N\cdot \nabla u_i^N) + \delta \Delta \rho _i^N u_i^N\big ) \cdot \nabla \log \rho _i^N dxds \nonumber \\&= -\varepsilon \int _0^t\int _{{\mathbb T}^d}\big (\partial _s u_i^N\cdot \nabla \rho _i^N + u_i^N\cdot \nabla u_i^N\cdot \nabla \rho _i^N + \delta \Delta \rho _i^N u_i^N\cdot \nabla \log \rho _i^N\big )dxds \nonumber \\&=: I_{11}+I_{12}+I_{13}. \end{aligned}$$

The term $I_{11}$ is rewritten according to

$$\begin{aligned} I_{11}&= -\varepsilon \int _0^t\int _{{\mathbb T}^d}\big (\partial _s(u_i^N\cdot \nabla \rho _i^N) - u_i^N\cdot \nabla \partial _s\rho _i^N\big ) dxds \\&= -\varepsilon \int _0^t\frac{d}{ds} \int _{{\mathbb T}^d}u_i^N\cdot \nabla \rho _i^N dxds - \varepsilon \int _0^t\int _{{\mathbb T}^d}\operatorname {div}u_i^N\big (-\operatorname {div}(\rho _i^N u_i^N) + \delta \Delta \rho _i^N\big ) dxds \\&= -\varepsilon \int _{{\mathbb T}^d}u_i^N(t)\cdot \nabla \rho _i^N(t) dx + \varepsilon \int _{{\mathbb T}^d}u_i^N(0)\cdot \nabla \rho _i^N(0)dx \\&\phantom {xx}+ \varepsilon \int _0^t\int _{{\mathbb T}^d} (u_i^N\cdot \nabla \rho _i^N)\operatorname {div}u_i^N dxds + \varepsilon \int _0^t\int _{{\mathbb T}^d}\rho _i^N(\operatorname {div}u_i^N)^2 dxds \\&\phantom {xx} - \delta \varepsilon \int _0^t\int _{{\mathbb T}^d}\Delta \rho _i^N \operatorname {div}u_i^N dxds =: I_{111}+\cdots +I_{115}. \end{aligned}$$

Corollary 7 shows that, for $0<t<T$,

$$\begin{aligned} I_{111} + I_{112}&= -\varepsilon \int _{{\mathbb T}^d}u_i^N(t)\cdot \nabla \rho _i^N(t)dx + \varepsilon \int _{{\mathbb T}^d}u_i^N(0)\cdot \nabla \rho _i^N(0)dx \\&\le 2\big (\sqrt{\varepsilon }\Vert (\rho _i^N)^{1/2}u_i^N \Vert _{L^\infty (0,T;L^2(\Omega ))}\big )\big (\sqrt{\varepsilon } \Vert \nabla (\rho _i^N)^{1/2}\Vert _{L^\infty (0,T;L^2({\mathbb T}^d))}\big ) \\&\phantom {xx} + 2\varepsilon \Vert (\rho _i^0)^{1/2}u_i^0\Vert _{L^2({\mathbb T}^d)} \Vert \nabla (\rho _i^0)^{1/2}\Vert _{L^2({\mathbb T}^d)} \le C, \\ I_{114}&\le \varepsilon \Vert (\rho _i^N)^{1/2}\nabla u_i^N\Vert _{L^2(0,T;L^2({\mathbb T}^d))}^2 \le C. \end{aligned}$$

Furthermore, using Hölder’s inequality, Corollary 7, and inequality (10),

$$\begin{aligned} I_{113}&\le 4\big (\root 4 \of {\varepsilon }\Vert (\rho _i^N)^{1/4}u_i^N \Vert _{L^4(0,T;L^4({\mathbb T}^d))}\big )\big (\sqrt{\varepsilon }\Vert (\rho _i^N)^{1/2} \nabla u_i^N\Vert _{L^2(0,T;L^2({\mathbb T}^d))}\big ) \\&\phantom {xx}\times \big (\root 4 \of {\varepsilon } \Vert \nabla (\rho _i^N)^{1/4}\Vert _{L^4(0,T;L^4({\mathbb T}^d))}\big ) \\&\le C + \frac{\varepsilon }{16c(d)} \int _0^t\int _{{\mathbb T}^d}|\nabla (\rho _i^N)^{1/4}|^4 dxds \\&\le C + \frac{\varepsilon }{16}\int _0^t\int _{{\mathbb T}^d} \rho _i^N|D^2\log \rho _i^N|^2 dxds, \end{aligned}$$

where in the last but one step we applied Young’s inequality. For the remaining term $I_{115}$, we first reformulate it, and then use similar arguments as for $I_{113}$:

$$\begin{aligned} I_{115}&= -\delta \varepsilon \int _0^t\int _{{\mathbb T}^d} \operatorname {div}(\rho _i^N\nabla \log \rho _i^N)\operatorname {div}u_i^N dxds \\&= -\delta \varepsilon \int _0^t\int _{{\mathbb T}^d}\big (\rho _i^N\Delta \log \rho _i^N + \nabla \rho _i^N\cdot \nabla \log \rho _i^N\big )\operatorname {div}u_i^N dxds \\&= -\delta \varepsilon \int _0^t\int _{{\mathbb T}^d}\big ((\rho _i^N)^{1/2} \Delta \log \rho _i^N + 16|\nabla (\rho _i^N)^{1/4}|^2\big )(\rho _i^N)^{1/2} \operatorname {div}u_i^N dxds \\&\le \delta \sqrt{\varepsilon }\big (\Vert (\rho _i^N)^{1/2}\Delta \log \rho _i^N \Vert _{L^2(0,T;L^2({\mathbb T}^d))} + 16\Vert \nabla (\rho _i^N)^{1/4}\Vert _{L^4(0,T;L^4({\mathbb T}^d))}^2\big ) \\&\phantom {xx}\times \sqrt{\varepsilon } \Vert (\rho _i^N)^{1/2}\nabla u_i^N\Vert _{L^2(0,T;L^2({\mathbb T}^d))} \\&\le C + \frac{\varepsilon }{16}\int _0^t\int _{{\mathbb T}^d} \rho _i^N|D^2\log \rho _i^N|^2 dxds. \end{aligned}$$

In a similar way, the bounds in Corollary 7 yield

$$\begin{aligned} I_{12}&\le 4\big (\root 4 \of {\varepsilon }\Vert (\rho _i^N)^{1/4}u_i^N \Vert _{L^4(0,T;L^4({\mathbb T}^d))}\big )\big (\sqrt{\varepsilon }\Vert (\rho _i^N)^{1/2} \nabla u_i^N\Vert _{L^2(0,T;L^2({\mathbb T}^d))}\big ) \\&\phantom {xx}\times \big (\root 4 \of {\varepsilon }\Vert \nabla (\rho _i^N)^{1/4} \Vert _{L^4(0,T;L^4({\mathbb T}^d))}\big ) \\&\le C + \frac{\varepsilon }{16}\int _0^t\int _{{\mathbb T}^d} \rho _i^N|D^2\log \rho _i^N|^2 dxds. \end{aligned}$$

We conclude from (22) that

$$\begin{aligned} I_1 \le C + \frac{3\varepsilon }{16}\int _0^t\int _{{\mathbb T}^d} \rho _i^N|D^2\log \rho _i^N|^2 dxds - \delta \varepsilon \int _0^t\int _{{\mathbb T}^d} \Delta \rho _i^N u_i^N\cdot \nabla \log \rho _i^N dxds, \end{aligned}$$

and the last term cancels with a part of $I_7$, since

$$\begin{aligned} I_7&= \delta \varepsilon \int _0^t\int _{{\mathbb T}^d}\big (\nabla \log \rho _i^N \cdot \nabla u_i^N\cdot \nabla \rho _i^N + \Delta \rho _i^Nu_i^N\cdot \nabla \log \rho _i^N\big )dxds \\&\le \sqrt{\delta }\big (4\root 4 \of {\delta \varepsilon }\Vert \nabla (\rho _i^N)^{1/4} \Vert _{L^4(0,T;L^4({\mathbb T}^d))}\big )^2\big ( \sqrt{\varepsilon }\Vert (\rho _i^N)^{1/2}\nabla u_i^N \Vert _{L^2(0,T;L^2({\mathbb T}^d))}\big ) \\&\phantom {xx}+ \delta \varepsilon \int _0^t\int _{{\mathbb T}^d} \Delta \rho _i^Nu_i^N\cdot \nabla \log \rho _i dxds \\&\le C + \delta \varepsilon \int _0^t\int _{{\mathbb T}^d} \Delta \rho _i^Nu_i^N\cdot \nabla \log \rho _i dxds. \end{aligned}$$

This shows that

23 $$\begin{aligned} I_1 + I_2 + I_7 \le C - \frac{5\varepsilon }{16} \int _0^t\int _{{\mathbb T}^d}\rho _i^N|D^2\log \rho _i^N|^2 dxds. \end{aligned}$$

*Step 2: Estimation of* $I_3,\ldots ,I_6$: We continue with the estimate of $I_3$:

$$\begin{aligned} I_3&= -\varepsilon \int _0^t\int _{{\mathbb T}^d} \rho _i^N\nabla u_i^N:D^2\log \rho _i^N dxds \\&\le \frac{\varepsilon }{16}\int _0^t\int _{{\mathbb T}^d}\rho _i^N|D^2\log \rho _i^N|^2 dxds + 4\varepsilon \int _0^t\int _{{\mathbb T}^d}\rho _i^N|\nabla u_i^N|^2 dxds \\&\le \frac{\varepsilon }{16}\int _0^t\int _{{\mathbb T}^d}\rho _i^N|D^2\log \rho _i^N|^2 dxds + C. \end{aligned}$$

By the approximative mass balance equation (16), we have

$$\begin{aligned} I_4&= -\varepsilon \int _0^t\int _{{\mathbb T}^d}\frac{u_i^N\cdot \nabla \rho _i^N}{\rho _i^N} dxds = \varepsilon \int _0^t\int _{{\mathbb T}^d}\frac{1}{\rho _i^N}\big (\partial _s\rho _i^N + \rho _i^N\operatorname {div}u_i^N - \delta \Delta \rho _i^N\big )dxds \\&= \varepsilon \int _0^t\int _{{\mathbb T}^d}\partial _s\log \rho _i^N dxds + \varepsilon \int _0^t\int _{{\mathbb T}^d}\operatorname {div}u_i^N dxds - \delta \varepsilon \int _0^t\int _{{\mathbb T}^d}|\nabla \log \rho _i^N|^2 dxds \\&= \varepsilon \int _{{\mathbb T}^d}\log \rho _i^N(t)dx - \varepsilon \int _{{\mathbb T}^d}\log \rho _i^N(0)dx - \delta \varepsilon \int _0^t\int _{{\mathbb T}^d}|\nabla \log \rho _i^N|^2 dxds \\&\le \varepsilon \int _{{\mathbb T}^d}(\rho _i^N(t)-1)dx - \varepsilon \int _{{\mathbb T}^d}\log \rho _i^0 dx - \delta \varepsilon \int _{{\mathbb T}^d}|\nabla \log \rho _i^N|^2 dxds. \end{aligned}$$

Because of the $L^2({\mathbb T}^d)$ bound for $\rho _i^N$ in Corollary 7, the first term on the right-hand side is bounded uniformly in $(N,\delta ,\varepsilon )$. The same holds true for the second term on the right-hand side, since $\log \rho _i^0$ is assumed to be integrable. We infer that

$$\begin{aligned} I_4 \le C - \delta \varepsilon \int _{{\mathbb T}^d}|\nabla \log \rho _i^N|^2 dxds. \end{aligned}$$

Furthermore, using Hölder’s inequality, Young’s inequality, and then inequality (10),

$$\begin{aligned} I_5&\le 4\big (\root 4 \of {\varepsilon }\Vert (\rho _i^N)^{1/4}u_i^N \Vert _{L^{4}(0,\infty ;L^{4}({\mathbb T}^d))}\big )^3 \big (\root 4 \of {\varepsilon }\Vert \nabla (\rho _i^N)^{1/4} \Vert _{L^4(0,\infty ;L^4({\mathbb T}^d))}\big ) \\&\le C + \frac{\varepsilon }{16}\int _0^t\int _{{\mathbb T}^d}\rho _i^N|D^2\log \rho _i^N|^2 dxds. \end{aligned}$$

Finally, the term $I_6$ is rewritten as

$$\begin{aligned} I_6 = -\int _0^t\int _{{\mathbb T}^d}\nabla \rho _i^N\cdot \nabla (\rho _1^N+\rho _2^N)dxds, \end{aligned}$$

and it becomes nonpositive when added for $i=1,2$. We conclude that

24 $$\begin{aligned} I_3+\cdots +I_6&\le C + \frac{\varepsilon }{8}\int _0^t\int _{{\mathbb T}^d} \rho _i^N|D^2\log \rho _i^N|^2 dxds - \delta \varepsilon \int _{{\mathbb T}^d}|\nabla \log \rho _i^N|^2 dxds \nonumber \\&\phantom {xx} -\int _0^t\int _{{\mathbb T}^d}\nabla \rho _i^N\cdot \nabla (\rho _1^N+\rho _2^N)dxds. \end{aligned}$$

*Step 3: End of the proof:* We insert (23) and (24) into (21) and sum over $i=1,2$ to conclude from (20) the desired entropy inequality. $\square$

The entropy inequality allows us to improve some bounds from Corollary 7.

#### Corollary 9

(Uniform estimates II). Let $(\rho ^N,u^N)$ be the local solution to (16)–(17) constructed in Section 2.1. Then there exists $C>0$ independent of $(\delta ,\varepsilon ,N)$ such that

$$\begin{aligned}&\Vert \nabla (\rho _1^N+\rho _2^N)\Vert _{L^2(0,\infty ;L^2({\mathbb T}^d))} \le C, \\&\sqrt{\varepsilon }\Vert (\rho _i^N)^{1/2}\Vert _{L^2(0,\infty ;H^2({\mathbb T}^d))} + \root 4 \of {\varepsilon }\Vert \nabla (\rho _i^N)^{1/4}\Vert _{L^4(0,\infty ;L^4({\mathbb T}^d))} \le C. \end{aligned}$$

### Further uniform estimates

We derive some spatial and time regularity bounds for $\rho _i^N$ and $\rho _i^N u_i^N$ uniform in *N*, which are needed for the limit $N\rightarrow \infty$.

#### Lemma 10

(Spatial regularity). For any $T>0$, there exists a constant $C(\varepsilon )>0$ independent of *N* and $\delta$ such that

$$\begin{aligned} \Vert \rho _i^N\Vert _{L^\infty (0,T;L^3({\mathbb T}^d))} + \Vert (\rho _i^N)^{1/2}\Vert _{L^4(0,T;W^{1,3}({\mathbb T}^d))}&\le C(\varepsilon ), \\ \Vert \rho _i^N\Vert _{L^2(0,T;W^{2,3/2}({\mathbb T}^d))} + \Vert \rho _i^N u_i^N\Vert _{L^2(0,T;W^{1,3/2}({\mathbb T}^d))}&\le C(\varepsilon ). \end{aligned}$$

#### Proof

The first bound for $\rho _i^N$ is an immediate consequence of Corollary 7 and the Sobolev embedding $H^1({\mathbb T}^d)\hookrightarrow L^6({\mathbb T}^d)$, yielding a uniform bound for $(\rho _i^N)^{1/2}$ in $L^\infty (0,T;L^6({\mathbb T}^d))$ (uniform in *N* and $\delta$). It follows from the embedding $H^2({\mathbb T}^d)\hookrightarrow L^\infty ({\mathbb T}^d)$ (here we use the condition $d\le 3$) and Corollary 9 that $(\rho _i^N)^{1/2}$ is uniformly bounded in $L^2(0,T;L^\infty ({\mathbb T}^d))$. Then, together with the uniform bound for $(\rho _i^N)^{1/2}u_i^N$ in $L^\infty (0,T;L^2({\mathbb T}^d))$ from Corollary 7, we obtain a uniform estimate for $\rho _i^Nu_i^N=(\rho _i^N)^{1/2}\cdot (\rho _i^N)^{1/2}u_i^N$ in $L^2(0,T;L^2({\mathbb T}^d))$. Furthermore, by Corollary 9, $(\nabla (\rho _i^N)^{1/2})$ is bounded in $L^2(0,T;L^6({\mathbb T}^d))$. Then

$$\begin{aligned} \nabla (\rho _i^N u_i^N) = 2\nabla (\rho _i^N)^{1/2}\cdot (\rho _i^N)^{1/2}u_i^N + 2(\rho _i^N)^{1/2}\cdot (\rho _i^N)^{1/2}\nabla u_i^N \end{aligned}$$

is uniformly bounded in $L^2(0,T;L^{3/2}({\mathbb T}^d))$. This yields a uniform bound for $\rho _i^Nu_i^N$ in $L^2(0,T;W^{1,3/2}({\mathbb T}^d))$.

Next, we apply the Gagliardo–Nirenberg inequality with $\theta =1/2$:

$$\begin{aligned} \Vert \nabla (\rho _i^N)^{1/2}\Vert _{L^4(0,T;L^3({\mathbb T}^d))}^4&\le C\int _0^T\Vert (\rho _i^N)^{1/2}\Vert _{H^2({\mathbb T}^d)}^{4\theta } \Vert (\rho _i^N)^{1/2}\Vert _{L^6({\mathbb T}^d)}^{4(1-\theta )}dt \\&\le C\Vert (\rho _i^N)^{1/2}\Vert _{L^\infty (0,T;L^6({\mathbb T}^d))}^{2} \int _0^T\Vert (\rho _i^N)^{1/2}\Vert _{H^2({\mathbb T}^d)}^2 dt \le C, \end{aligned}$$

showing that $(\rho _i^N)^{1/2}$ is uniformly bounded in $L^4(0,T;W^{1,3}({\mathbb T}^d))$. Because of inequality (10), the uniform bound for $(\rho _i^N)^{1/2}D^2\log \rho _i^N$ in $L^2(0,T;L^2({\mathbb T}^d))$ implies a uniform bound for $D^2(\rho _i^N)^{1/2}$ in $L^2(0,T;$ $L^2({\mathbb T}^d))$. Thus,

$$\begin{aligned} D^2\rho _i^N = 2(\rho _i^N)^{1/2}D^2(\rho _i^N)^{1/2} + 2\nabla (\rho _i^N)^{1/2}\otimes \nabla (\rho _i^N)^{1/2} \end{aligned}$$

is uniformly bounded in $L^2(0,T;L^{3/2}({\mathbb T}^d))$. Finally, the bound for $(\rho _i^N)^{1/4}$ in $L^\infty (0,T;$ $L^{12}({\mathbb T}^d))$ and for $\nabla (\rho _i^N)^{1/4}$ in $L^4(0,T;L^4({\mathbb T}^d))$ yield an estimate for $\nabla (\rho _i^N)^{1/2}=2(\rho _i^N)^{1/4}$ $\nabla (\rho _i^N)^{1/4}$ in $L^4(0,T;L^3({\mathbb T}^d))$, finishing the proof. $\square$

#### Lemma 11

(Time regularity). For any $T>0$, there exists a constant $C(\varepsilon )>0$ independent of *N* and $\delta$ such that for $s>d/2+1$,

$$\begin{aligned} \Vert \partial _t\rho _i^N\Vert _{L^2(0,T;L^{3/2}({\mathbb T}^d))} + \Vert \partial _t(\rho _i^Nu_i^N)\Vert _{L^{4/3}(0,T;H^{s}({\mathbb T}^d)')} + \Vert \partial _t(\rho _i^N)^{1/2}\Vert _{L^2(0,T;L^{2}({\mathbb T}^d))} \le C. \end{aligned}$$

#### Proof

We deduce from Lemma 10 that

$$\begin{aligned} \partial _t\rho _i^N = -\operatorname {div}(\rho _i^Nu_i^N) + \delta \Delta \rho _i^N \end{aligned}$$

is uniformly bounded in $L^2(0,T;L^{3/2}({\mathbb T}^d))$. The estimate on $\partial _t(\rho _i^Nu_i^N)$ follows from the following bounds, which are consequences of Corollaries 7 and 9, as well as from the spatial regularity of Lemma 10:

- The sequence $(\rho _i^N u_i^N\otimes u_i^N)$ is bounded in $L^\infty (0,T;L^1({\mathbb T}^d))$. Hence, $\operatorname {div}(\rho _i^N u_i^N\otimes u_i^N)$ is bounded in $L^\infty (0,T;H^s({\mathbb T}^d)')$, since $H^s({\mathbb T}^d)$ $\hookrightarrow W^{1,\infty }({\mathbb T}^d)$ for $s>d/2+1$.
- We know that $\rho _i^N\nabla u_i^N =(\rho _i^N)^{1/2}\cdot (\rho _i^N)^{1/2}\nabla u_i^N$ is bounded in $L^2(0,T;L^{3/2}({\mathbb T}^d))$. Thus, $\operatorname {div}(\rho _i^N\nabla u_i^N)$ is bounded in $L^2(0,T;W^{1,3}({\mathbb T}^d)')\hookrightarrow L^2(0,T;H^s({\mathbb T}^d)')$.
- Let $\psi \in L^4(0,T;W^{1,3}({\mathbb T}^d;{\mathbb R}^d))$. Then, by integration by parts,
  $$\begin{aligned} \int _0^T&\int _{{\mathbb T}^d}\rho _i^N\nabla \bigg (\frac{\Delta (\rho _i^N)^{1/2}}{ (\rho _i^N)^{1/2}}\bigg )\cdot \psi dxds \\&= -\int _0^T\int _{{\mathbb T}^d}\Delta (\rho _i^N)^{1/2}\big (2\nabla (\rho _i^N)^{1/2} \cdot \psi + (\rho _i^N)^{1/2}\operatorname {div}\psi \big )dxds \\&\le 2\Vert \Delta (\rho _i^N)^{1/2}\Vert _{L^2(0,T;L^2({\mathbb T}^d))} \Vert \nabla (\rho _i^N)^{1/2}\Vert _{L^4(0,T;L^{3}({\mathbb T}^d))} \Vert \psi \Vert _{L^4(0,T;L^6({\mathbb T}^d))} \\&\phantom {xx} + \Vert \Delta (\rho _i^N)^{1/2}\Vert _{L^2(0,T;L^2({\mathbb T}^d))} \Vert (\rho _i^N)^{1/2}\Vert _{L^\infty (0,T;L^6({\mathbb T}^d))} \Vert \psi \Vert _{L^2(0,T;W^{1,3}({\mathbb T}^d))} \\&\le C\Vert \psi \Vert _{L^4(0,T;W^{1,3}({\mathbb T}^d))}. \end{aligned}$$
  This proves that $\rho _i^N\nabla (\Delta (\rho _i^N)^{1/2}/(\rho _i^N)^{1/2})$ is uniformly bounded in $L^{4/3}(0,T;W^{1,3}({\mathbb T}^d)')$ $\hookrightarrow L^{4/3}(0,T;H^s({\mathbb T}^d)')$.
- The sequence $\rho _i^N|u_i^N|^2 u_i^N = (\rho _i^N)^{1/2}u_i^N\cdot (\rho _i^N)^{1/2}|u_i^N|^2$ is bounded in $L^2(0,T;L^1({\mathbb T}^d))$ $\hookrightarrow L^{2}(0,T;H^s({\mathbb T}^d)')$, since $(\rho _i^N)^{1/2}u_i^N$ is bounded in $L^\infty (0,T;L^2({\mathbb T}^d))$ and $(\rho _i^N)^{1/2}$ $|u_i^N|^2$ is bounded in $L^2(0,T;L^2({\mathbb T}^d))$ by Corollary 7.
- By Lemma 10, $\rho _i^N u_i^N$ is uniformly bounded in $L^2(0,T;W^{1,3/2}({\mathbb T}^d))\hookrightarrow L^2(0,T;$ $L^{3/2}({\mathbb T}^d))$.
- In view of the $L^\infty (0,T;L^3({\mathbb T}^d))$ bound of $\rho _i^N$ from Lemma 10 and the $L^2(0,T;L^2({\mathbb T}^d))$ bound of $\nabla (\rho _1^N+\rho _2^N)$ from Corollary 9, $\rho _i^N\nabla (\rho _1^N+\rho _2^N)$ is bounded in $L^2(0,T;L^{6/5}({\mathbb T}^d))$ $\hookrightarrow L^2(0,T;H^s({\mathbb T}^d)')$.
- We deduce from the $L^2(0,T;L^2({\mathbb T}^d))$ bound of $(\rho _i^N)^{1/2}u_i^N$ (Corollary 7) and the $L^4(0,T;L^3({\mathbb T}^d))$ bound of $\nabla (\rho _i^N)^{1/2}$ that $u_i^N\otimes \nabla \rho _i^N=2(\rho _i^N)^{1/2}u_i^N\otimes \nabla (\rho _i^N)^{1/2}$ is bounded in $L^{4/3}(0,T;L^{6/5}({\mathbb T}^d))$. Hence, the sequence $\operatorname {div}(u_i^N\otimes \nabla \rho _i^N)$ is bounded in $L^{4/3}(0,T;W^{1,6}({\mathbb T}^d)')\hookrightarrow L^{4/3}(0,T;H^s({\mathbb T}^d)')$.

We conclude that

$$\begin{aligned} \partial _t(\rho _i^Nu_i^N)&= -\operatorname {div}(\rho _i^Nu_i^N\otimes u_i^N) + \rho _i^N\nabla \bigg (\frac{\Delta (\rho _i^N)^{1/2}}{ (\rho _i^N)^{1/2}} \bigg ) + \operatorname {div}(\rho _i^N\nabla u_i^N) - u_i^N \\&\phantom {xx}- \rho _i^N u_i^N|u_i^N|^2 - \varepsilon ^{-1}k_i^{-1}\rho _i^Nu_i^N - \varepsilon ^{-1}\rho _i^N\nabla (\rho _1+\rho _2^N) + \delta \operatorname {div}(u_i^N\otimes \nabla \rho _i^N) \end{aligned}$$

is uniformly bounded in $L^{4/3}(0,T;H^s({\mathbb T}^d)')$. Finally, the sequence

$$\begin{aligned} \partial _t(\rho _i^N)^{1/2}&= -\frac{1}{2}(\rho _i^N)^{1/2}\operatorname {div}u_i^N - 2\nabla (\rho _i^N)^{1/4}\cdot ((\rho _i^N)^{1/4}u_i^N) \\&\phantom {xx}+ \delta \Delta (\rho _i^N)^{1/2} + 4\delta |\nabla (\rho _i^N)^{1/4}|^2 \end{aligned}$$

is bounded in $L^2(0,T;L^2({\mathbb T}^d))$. $\square$

### Limit $N\rightarrow \infty$

The spatial and time regularity for $(\rho _i^N)^{1/2}$, $\rho _i^N$, and $\rho _i^Nu_i^N$ allow us to apply the Aubin–Lions compactness lemma to conclude the existence of a subsequence (not relabeled) such that, as $N\rightarrow \infty$,

$$\begin{aligned} (\rho _i^N)^{1/2}\rightarrow \sqrt{\rho _i}&\quad \text{ strongly } \text{ in } L^2(0,T;H^1({\mathbb T}^d)), \\ \rho _i^N\rightarrow \rho _i&\quad \text{ strongly } \text{ in } L^2(0,T;L^2({\mathbb T}^d)), \\ \rho _i^Nu_i^N \rightarrow J_i&\quad \text{ strongly } \text{ in } L^2(0,T;L^2({\mathbb T}^d)). \end{aligned}$$

Furthermore, we have the weak convergences (up to subsequences)

$$\begin{aligned} (\rho _i^N)^{1/2}\rightharpoonup \sqrt{\rho _i}&\quad \text{ weakly } \text{ in } L^2(0,T;H^2({\mathbb T}^d)), \\ u_i^N\rightharpoonup u_i&\quad \text{ weakly } \text{ in } L^2(0,T;L^2({\mathbb T}^d)). \end{aligned}$$

It follows that $\rho _i^N u_i^N\rightharpoonup \rho _iu_i$ weakly in $L^1(0,T;L^1({\mathbb T}^d))$, showing that $J_i=\rho _iu_i$. Moreover, by Lemma 10, $\delta \nabla \rho _i^N=2\delta (\rho _i^N)^{1/2}\nabla (\rho _i^N)^{1/2}\rightarrow 0$ strongly in $L^4(0,T;L^2({\mathbb T}^d))$.

With these convergences, we can pass to the limit $N\rightarrow \infty$ and $\delta \rightarrow 0$ in (16), formulated for $\phi \in C_0^\infty ({\mathbb T}^d\times [0,T))$ as

$$\begin{aligned} 0&= -\int _0^T\int _{{\mathbb T}^d}\rho _i^N\partial _t\phi dxdt - \int _{{\mathbb T}^d}\rho _i^0\phi (0)dx - \int _0^T\int _{{\mathbb T}^d}\rho _i^N u_i^N\cdot \nabla \phi dxdt \\&\phantom {xx}+ \delta \int _0^T\int _{{\mathbb T}^d}\nabla \rho _i^N\cdot \nabla \phi dxdt, \end{aligned}$$

leading to

$$\begin{aligned} 0 = -\int _0^T\int _{{\mathbb T}^d}\rho _i\partial _t\phi dxdt - \int _{{\mathbb T}^d}\rho _i^0\phi (0)dx - \int _0^T\int _{{\mathbb T}^d}\rho _i u_i\cdot \nabla \phi dxdt. \end{aligned}$$

The limit in the momentum balance equation (17) is more involved. The strong convergence of $\rho _i^Nu_i^N$ and the weak convergence of $u_i^N$ in $L^2(0,T;L^2({\mathbb T}^d))$ lead to

$$\begin{aligned} \rho _i^Nu_i^N\otimes u_i^N\rightharpoonup \rho _iu_i\otimes u_i \quad \text{ weakly } \text{ in } L^1(0,T;L^1({\mathbb T}^d)). \end{aligned}$$

Similarly, $(\rho _i^N)^{1/2}u_i^N\rightharpoonup \sqrt{\rho _i}u_i$ weakly in $L^2(0,T;L^2({\mathbb T}^d))$, taking into account Corollary 7. Then, together with the strong convergences of $\nabla (\rho _i^N)^{1/2}$ and $\rho _i^Nu_i^N$ in $L^2(0,T;L^2({\mathbb T}^d))$, we find that for $\psi \in C_0^\infty ({\mathbb T}^d\times (0,T);{\mathbb R}^{d\times d}))$, integrating by parts,

25 $$\begin{aligned} -\int _0^T&\int _{{\mathbb T}^d}\rho _i^N\nabla u_i^N:\psi dxdt = \int _0^T\int _{{\mathbb T}^d}u_i^N\cdot \operatorname {div}(\rho _i^N\psi )dxdt \nonumber \\&= \int _0^T\int _{{\mathbb T}^d}\big (2(\rho _i^N)^{1/2}u_i^N\cdot \psi \cdot \nabla (\rho _i^N)^{1/2} + \rho _i^Nu_i^N\cdot \operatorname {div}\psi \big )dxdt \nonumber \\&\rightarrow \int _0^T\int _{{\mathbb T}^d}\big (2\sqrt{\rho _i}u_i\cdot \psi \cdot \nabla \sqrt{\rho _i} + \rho _iu_i\cdot \operatorname {div}\psi \big )dxdt \nonumber \\&= -\int _0^T\langle \nabla u_i,\rho _i\psi \rangle _{H^1({\mathbb T}^d)', H^1({\mathbb T}^d)}dt = -\int _0^T\langle \rho _i\nabla u_i,\psi \rangle _{ \mathcal {D}'({\mathbb T}^d),\mathcal {D}({\mathbb T}^d)}dt. \end{aligned}$$

Thus, $\operatorname {div}(\rho _i^N\nabla u_i^N)\rightarrow \operatorname {div}(\rho _i\nabla u_i)$ in the sense of distributions. In fact, because of the uniform bounds of $(\rho _i^N)^{1/2}$ in $L^\infty (0,T;H^1({\mathbb T}^d))\hookrightarrow L^\infty (0,T;L^6({\mathbb T}^d))$ and of $(\rho _i^N)^{1/2}\nabla u_i^N$ in $L^2(0,T;L^2({\mathbb T}^d))$, this convergence also holds in the weak topology of $L^2(0,T;L^{3/2}({\mathbb T}^d))$. The same bounds imply that

$$\begin{aligned} u_i^N\otimes \nabla \rho _i^N = 2(\rho _i^N)^{1/2}u_i^N\otimes \nabla (\rho _i^N)^{1/2} \rightharpoonup 2\sqrt{\rho _i}u_i\otimes \nabla \sqrt{\rho _i} = u_i\otimes \nabla \rho _i \end{aligned}$$

weakly in $L^1(0,T;L^1({\mathbb T}^d))$ and hence $\operatorname {div}(u_i^N\otimes \nabla \rho _i^N)\rightharpoonup \operatorname {div}(u_i\otimes \nabla \rho _i)$ weakly in $L^1(0,T;$ $H^s({\mathbb T}^d)')$.

Next, since $\Delta (\rho _i^N)^{1/2}\rightharpoonup \Delta \sqrt{\rho _i}$ weakly in $L^2(0,T;L^2({\mathbb T}^d))$ and $\nabla (\rho _i^N)^{1/2}\rightarrow \nabla \sqrt{\rho _i}$ strongly in $L^2(0,T;L^2({\mathbb T}^d))$, for $\psi \in C_0^\infty ({\mathbb T}^d\times (0,T);{\mathbb R}^d)$,

$$\begin{aligned} \int _0^T\int _{{\mathbb T}^d}&\rho _i^N\nabla \bigg (\frac{\Delta (\rho _i^N)^{1/2}}{ (\rho _i^N)^{1/2}}\bigg )\cdot \psi dxdt \\&= -\int _0^T\int _{{\mathbb T}^d}\Delta (\rho _i^N)^{1/2}\big (2\nabla (\rho _i^N)^{1/2} \cdot \phi + (\rho _i^N)^{1/2}\operatorname {div}\psi \big )dxdt \\&\rightarrow -\int _0^T\int _{{\mathbb T}^d}\Delta \sqrt{\rho _i}\big (2\nabla \sqrt{\rho _i} \cdot \psi + \sqrt{\rho _i}\operatorname {div}\psi \big )dxdt. \end{aligned}$$

The convergence $\rho _i^N|u_i^N|^2u_i^N\rightarrow \rho _i|u_i|^2u_i$ strongly in $L^1(0,T;L^1({\mathbb T}^d))$ has been proved in [35, Lemma 2.3]. For completeness, we recall the proof. The strong convergences of $(\rho _i^N)$ and $(\rho _i^Nu_i^N)$ imply, up to subsequence, that $\rho _i^N\rightarrow \rho _i$ and $\rho _i^Nu_i^N\rightarrow \rho _iu_i$ a.e. Hence, for a.e. (*x*, *t*), $u_i^N=(\rho _i^Nu_i^N)/\rho _i^N\rightarrow u_i$ whenever $\rho _i^N(x,t)\ne 0$. For a.e. (*x*, *t*) for which $\rho _i^N(x,t)=0$, we have

$$\begin{aligned} g_i^N := \rho _i^N|u_i^N|^2u_i^N\textrm{1}_{\{|u_i^N|\le M\}} \le \rho _I^NM^3 = 0 \end{aligned}$$

for any $M>0$. Consequently, $g_i^N\rightarrow \rho _i|u_i|^2u_i\textrm{1}_{\{|u_i|\le M\}}$ a.e. As the sequence $(\rho _i^N)$ is bounded in $L^\infty (0,T;L^2({\mathbb T}^d))$, $g_i^N$ is bounded in the same space. Then dominated convergence implies that

26 $$\begin{aligned} g_i^N\rightarrow \rho _i|u_i|^2u_i\textrm{1}_{\{|u_i|\le M\}} \quad \text{ strongly } \text{ in } L^1(0,T;L^1({\mathbb T}^d)). \end{aligned}$$

Now, for any $M>0$,

$$\begin{aligned} \int _0^T&\int _{{\mathbb T}^d}\big |\rho _i^N|u_i^N|^2u_i^N - \rho |u_i|^2u_i\big |dxdt \\&\le \int _0^T\int _{{\mathbb T}^d} \big |\rho _i^N|u_i^N|^2u_i^N\textrm{1}_{\{|u_i^N|\le M\}} - \rho _i|u_i|^2u_i\textrm{1}_{\{|u_i|\le M\}}\big |dxdt \\&\phantom {xx}+ \int _0^T\int _{{\mathbb T}^d} \big (\rho _i^N|u_i^N|^3\textrm{1}_{\{|u_i^N|>M\}} + \rho _i|u_i|^3\textrm{1}_{\{|u_i|>M\}}\big )dxdt \\&\le \int _0^T\int _{{\mathbb T}^d} \big |g_i^N - \rho _i|u_i|^2u_i \textrm{1}_{\{|u_i|\le M\}}\big |dxdt \\&\phantom {xx}+ \frac{1}{M}\int _0^T\int _{{\mathbb T}^d}\big ( \rho _i^N|u_i^N|^4 + \rho _i|u_i|^4\big )dxdt, \end{aligned}$$

observing that $\rho _i|u_i|^4$ is an element of $L^1(0,T;L^1({\mathbb T}^d))$. The convergence (26) shows that

$$\begin{aligned} \lim _{N\rightarrow \infty }\int _0^T\int _{{\mathbb T}^d}\big |\rho _i^N|u_i^N|^2u_i^N - \rho |u_i|^2u_i\big |dxdt \le \frac{C}{M} \end{aligned}$$

for some $C>0$ and for all $M>0$. The limit $M\rightarrow \infty$ finishes the proof of the strong convergence of $\rho _i^N|u_i^N|^2u_i^N$.

The weak convergence of $\nabla (\rho _1^N+\rho _2^N)$ and the strong convergence of $\rho _i^N$ in $L^2(0,T;L^2({\mathbb T}^d))$ imply that

$$\begin{aligned} \rho _i^N\nabla (\rho _1^N+\rho _2^N)\rightharpoonup \rho _i\nabla (\rho _1+\rho _2) \quad \text{ weakly } \text{ in } L^1(0,T;L^1({\mathbb T}^d)). \end{aligned}$$

We treat the $\delta$-regularized terms:

$$\begin{aligned}&\bigg |\delta \int _0^T\int _{{\mathbb T}^d}\nabla \rho _i^N\cdot \nabla \phi dxt\bigg | \le \delta \Vert \rho _i^N\Vert _{L^1(0,T;L^1({\mathbb T}^d))} \Vert \Delta \phi \Vert _{L^\infty (0,T;L^\infty ({\mathbb T}^d))} \rightarrow 0, \\&\bigg |\delta \varepsilon \int _0^T\int _{{\mathbb T}^d}(u_i^N\otimes \nabla \rho _i^N) :\nabla \phi dxdt\bigg | \le 2\delta \varepsilon \Vert (\rho _i^N)^{1/2}u_i^N \Vert _{L^2(0,T;L^2({\mathbb T}^d))} \\&\quad \phantom {xx}\times \Vert \nabla (\rho _i^N)^{1/2}\Vert _{L^2(0,T;L^2({\mathbb T}^d))} \Vert \nabla \phi \Vert _{L^\infty (0,T;L^\infty ({\mathbb T}^d))} \rightarrow 0 \end{aligned}$$

as $N\rightarrow \infty$ and $\delta \rightarrow 0$. These convergences are sufficient to pass to the limit $N\rightarrow \infty$ and $\delta \rightarrow 0$ in (17).

It remains to perform the limit in the approximate energy inequality (see Lemma 6). This follows from the weak lower semicontinuity of the norms if we show that

$$\begin{aligned} (\rho _i^N)^{1/2}\nabla u_i^N\rightharpoonup \sqrt{\rho _i}\nabla u_i&\quad \text{ weakly } \text{ in } L^2(0,T;L^2({\mathbb T}^d)), \\ (\rho _i^N)^{1/4}u_i^N \rightharpoonup \root 4 \of {\rho _i}u_i&\quad \text{ weakly } \text{ in } L^4(0,T;L^4({\mathbb T}^d)). \end{aligned}$$

In fact, we deduce from the bound on $(\rho _i^N)^{1/4}u_i^N$ that $(\rho _i^N)^{1/4}u_i^N\rightharpoonup y_i$ weakly in $L^4(0,T;$ $L^4({\mathbb T}^d))$ for some $y_i$. The strong convergence of $(\rho _i^N)^{1/4}$ in $L^4(0,T;L^4({\mathbb T}^d))$ and the weak convergence of $u_i^N$ in $L^2(0,T;L^2({\mathbb T}^d))$ imply that $(\rho _i^N)^{1/4}u_i^N\rightharpoonup \root 4 \of {\rho _i}u_i$ weakly in $L^{4/3}(0,T;$ $L^{4/3}({\mathbb T}^d))$ and consequently $y_i=\root 4 \of {\rho _i}u_i$. The remaining limit has been shown in (25).

## Proof of Theorem 2

We want to pass to the limit $\varepsilon \rightarrow 0$ in (5)–(6). Let $(\rho _i^\varepsilon ,u_i^\varepsilon )_{i=1,2}$ be a weak solution to (5)–(6) constructed in Theorem 1. We set $\rho ^\varepsilon =(\rho _1^\varepsilon ,\rho _2^\varepsilon )$, $u^\varepsilon =(u_1^\varepsilon ,u_2^\varepsilon )$, and $\bar{\rho }^\varepsilon =\rho _1^\varepsilon +\rho _2^\varepsilon$. The energy and entropy estimates from Corollaries 7 and 9 yield in the limit $N\rightarrow \infty$ and $\delta \rightarrow 0$ the existence of a constant $C>0$ independent of $\varepsilon$ such that for $i=1,2$,

27 $$\begin{aligned} \begin{aligned} \Vert \rho _i^\varepsilon \Vert _{L^\infty (0,T;L^2({\mathbb T}^d))} + \Vert \bar{\rho }^\varepsilon \Vert _{L^2(0,T;H^1({\mathbb T}^d))} + \Vert (\rho _i^\varepsilon )^{1/2}u_i^\varepsilon \Vert _{L^2(0,T;L^2({\mathbb T}^d))}&\le C, \\ \sqrt{\varepsilon }\Vert (\rho _i^\varepsilon )^{1/2}\Vert _{L^2(0,T;H^2({\mathbb T}^d))} + \root 4 \of {\varepsilon }\Vert (\rho _i^\varepsilon )^{1/4}\Vert _{L^4(0,T;W^{1,4}({\mathbb T}^d))}&\le C, \\ \sqrt{\varepsilon }\Vert u_i^\varepsilon \Vert _{L^2(0,T;L^2({\mathbb T}^d))} + \root 4 \of {\varepsilon }\Vert (\rho _i^\varepsilon )^{1/4}u_i^\varepsilon \Vert _{L^4(0,T;L^4({\mathbb T}^d))}&\le C. \end{aligned} \end{aligned}$$

This shows that $\rho _i^\varepsilon u_i^\varepsilon = (\rho _i^\varepsilon )^{1/2}\cdot (\rho _i^\varepsilon )^{1/2}u_i^\varepsilon$ is uniformly bounded in $L^2(0,T;L^{4/3}({\mathbb T}^d))$. Therefore, there exist subsequences (not relabeled) such that, as $\varepsilon \rightarrow 0$,

$$\begin{aligned} \rho _i^\varepsilon \rightharpoonup \rho _i&\quad \text{ weakly* } \text{ in } L^\infty (0,T;L^2({\mathbb T}^d)), \\ \rho _i^\varepsilon u_i^\varepsilon \rightharpoonup J_i&\quad \text{ weakly } \text{ in } L^2(0,T;L^{4/3}({\mathbb T}^d)),\ i=1,2. \end{aligned}$$

In particular, $\partial _t\bar{\rho }^\varepsilon = -\operatorname {div}(\rho _1^\varepsilon u_1^\varepsilon + \rho _2^\varepsilon u_2^\varepsilon )$ is uniformly bounded in $L^2(0,T;W^{1,4}({\mathbb T}^d)')$. Thanks to the $L^2(0,T;H^1({\mathbb T}^d))$ bound for $\bar{\rho }^\varepsilon$, we can apply the Aubin–Lions compactness lemma to conclude that, up to a subsequence,

$$\begin{aligned} \bar{\rho }^\varepsilon \rightarrow \bar{\rho }\quad \text{ strongly } \text{ in } L^2(0,T;L^p({\mathbb T}^d)), \ p<6. \end{aligned}$$

Then the limit in the sum of (5) over $i=1,2$, namely $\partial _t\bar{\rho }^\varepsilon +\operatorname {div}(\rho _1^\varepsilon u_1^\varepsilon + \rho _2^\varepsilon u_2^\varepsilon )=0$, shows that $\bar{\rho }$ solves

28 $$\begin{aligned} \partial _t\bar{\rho }+ \operatorname {div}(J_1+J_2) = 0. \end{aligned}$$

The limit in (6) is more involved, and we perform the limit only in the sum over $i=1,2$. We treat the various expressions term by term. First, the sequence

$$\begin{aligned} \rho _i^\varepsilon u_i^\varepsilon \otimes u_i^\varepsilon = \big ((\rho _i^\varepsilon )^{1/2}u_i^\varepsilon \big )\otimes \big ((\rho _i^\varepsilon )^{1/2}u_i^\varepsilon \big ) \end{aligned}$$

is bounded in $L^1(0,T;L^1({\mathbb T}^d))$, which implies that

$$\begin{aligned} \varepsilon \operatorname {div}(\rho _i^\varepsilon u_i^\varepsilon \otimes u_i^\varepsilon )\rightharpoonup 0 \quad \text{ weakly } \text{ in } L^1(0,T;H^s({\mathbb T}^d)'). \end{aligned}$$

Furthermore, again by (27),

$$\begin{aligned} \sqrt{\varepsilon }\rho _i^\varepsilon \nabla u_i^\varepsilon = \sqrt{\varepsilon }\nabla (\rho _i^\varepsilon u_i^\varepsilon ) - 2\sqrt{\varepsilon }((\rho _i^\varepsilon )^{1/2}u_i^\varepsilon ) \otimes \nabla (\rho _i^\varepsilon )^{1/2} \end{aligned}$$

is uniformly bounded in $L^2(0,T;W^{1,4}({\mathbb T}^d)')+L^1(0,T;L^1({\mathbb T}^d))$, and thus

$$\begin{aligned} \varepsilon \operatorname {div}(\rho _i^\varepsilon \nabla u_i^\varepsilon )\rightharpoonup 0\quad \text{ weakly } \text{ in } L^1(0,T;W^{2,4}({\mathbb T}^d)'). \end{aligned}$$

The Korteweg regularization in its weak formulation is estimated as follows:

$$\begin{aligned} \bigg |\varepsilon&\int _0^T\int _{{\mathbb T}^d}\Delta (\rho _i^\varepsilon )^{1/2}\big ( 4(\rho _i^\varepsilon )^{1/4}\nabla (\rho _i^\varepsilon )^{1/4}\cdot \psi + (\rho _i^\varepsilon )^{1/2}\operatorname {div}\psi \big )dxdt\bigg | \\&\le \root 4 \of {\varepsilon }\cdot \sqrt{\varepsilon }\Vert \Delta (\rho _i^\varepsilon )^{1/2}\Vert _{L^2(0,T;L^2({\mathbb T}^d))} \big (4\root 4 \of {\varepsilon }\Vert \nabla (\rho _i^\varepsilon )^{1/4}\Vert _{L^4(0,T;L^4({\mathbb T}^d))} \\&\phantom {xx}\times \Vert (\rho _i^\varepsilon )^{1/4}\Vert _{L^\infty (0,T;L^8({\mathbb T}^d))} \Vert \psi \Vert _{L^4(0,T;L^8({\mathbb T}^d))} \\&\phantom {xx}+ \root 4 \of {\varepsilon } \Vert (\rho _i^\varepsilon )^{1/2}\Vert _{L^\infty (0,T;L^4({\mathbb T}^d))} \Vert \operatorname {div}\psi \Vert _{L^2(0,T;L^4({\mathbb T}^d))}\big ) \le \root 4 \of {\varepsilon }C\rightarrow 0, \end{aligned}$$

where $\psi \in L^4(0,T;W^{1,4}({\mathbb T}^d;{\mathbb R}^d))$. The drag forces also vanish in the limit since $\varepsilon u_i^\varepsilon \rightarrow 0$ strongly in $L^2(0,T;L^2({\mathbb T}^d))$ and

$$\begin{aligned} \varepsilon \rho _i^\varepsilon |u_i^\varepsilon |^2 u_i^\varepsilon = \root 4 \of {\varepsilon }(\rho _i^\varepsilon )^{1/4}\big (\root 4 \of {\varepsilon } (\rho _i^\varepsilon )^{1/4}|u_i^\varepsilon |\big )^2\big (\root 4 \of {\varepsilon } (\rho _i^\varepsilon )^{1/4}u_i^\varepsilon ) = O(\root 4 \of {\varepsilon }) \rightarrow 0 \end{aligned}$$

strongly in $L^{4/3}(0,T;L^{8/7}({\mathbb T}^d))$. Finally,

$$\begin{aligned} \sum _{i=1}^2\rho _i^\varepsilon \nabla \bar{\rho }^\varepsilon = \bar{\rho }^\varepsilon \nabla \bar{\rho }^\varepsilon \rightharpoonup \bar{\rho }\nabla \bar{\rho }\quad \text{ weakly } \text{ in } L^1(0,T;L^{1}({\mathbb T}^d)). \end{aligned}$$

Hence, passing to the limit $\varepsilon \rightarrow 0$ in sum of the weak formulation (13) over $i=1,2$, we find that

$$ k_1^{-1}J_1 + k_2^{-1}J_2 = -\bar{\rho }\nabla \bar{\rho }, $$

or $J_1 = -k_1\bar{\rho }\nabla \bar{\rho }- (k_1/k_2)J_2$. Together with (28), $\bar{\rho }$ solves

$$\begin{aligned} \partial _t\bar{\rho }= k_1\operatorname {div}(\bar{\rho }\nabla \bar{\rho }) - \bigg (1-\frac{k_1}{k_2}\bigg )\operatorname {div}J_2 = \operatorname {div}\bigg (k_1\bar{\rho }\nabla \bar{\rho }- \bigg (1-\frac{k_1}{k_2}\bigg )J_2\bigg ). \end{aligned}$$

### Remark 12

If we can identify $J_2=-k_2\rho _2\nabla \bar{\rho }$, we obtain

$$\begin{aligned} k_1\bar{\rho }\nabla \bar{\rho }+ \bigg (1-\frac{k_1}{k_2}\bigg )k_2\rho _2\nabla \bar{\rho }= (k_1\rho _1+k_2\rho _2)\nabla \bar{\rho }, \end{aligned}$$

such that the limit equation becomes

$$\begin{aligned} \partial _t\bar{\rho }+ \operatorname {div}\big ((k_1\rho _1+k_2\rho _2)\nabla \bar{\rho }\big ) = 0. \end{aligned}$$

## Proof of Theorem 3: Relative Entropy Inequality

The solution $(\bar{\rho },\bar{u})$ with $\bar{u}=-\nabla \bar{\rho }$ is shown to solve fluid-type equations for which we derive an associated energy equality. Then we derive an inequality for the difference of the mass and momentum balance equations for $(\rho ^\varepsilon ,u^\varepsilon )$ and $(\bar{\rho },\bar{u})$. These results allow us to prove the relative entropy inequality and to finish the proof of Theorem 3.

### Energy equality for the limit system

We analyze the limit system for $\bar{\rho }=\rho _1+\rho _2$ and $\bar{u}=-\nabla \bar{\rho }$. The mass balance equation reads as

29 $$\begin{aligned} \partial _t\bar{\rho }+ \operatorname {div}(\bar{\rho }\bar{u}) = 0, \end{aligned}$$

with initial condition $\bar{\rho }(0)=\bar{\rho }^0:=\rho _1^0+\rho _2^0$. Inserting (29), the analog of the momentum balance equation for $\bar{u}$ becomes

30 $$\begin{aligned} \partial _t(\bar{\rho }\bar{u}) + \operatorname {div}(\bar{\rho }\bar{u}\otimes \bar{u}) = \bar{\rho }(\partial _t\bar{u}+(\bar{u}\cdot \nabla )\bar{u}) = \bar{\rho }\bar{e}, \end{aligned}$$

where $\bar{e}:=\partial _t\bar{u}+(\bar{u}\cdot \nabla )\bar{u}$ is the material derivative of $\bar{u}$. The initial condition is $\bar{\rho }(0)\bar{u}(0)=-\bar{\rho }^0\nabla \bar{\rho }^0$. According to Lemma 5, the weak formulations of (29)–(30) can be written as

31 $$\begin{aligned} 0&= -\int _0^t\int _{{\mathbb T}^d}\bar{\rho }\partial _s\phi dxds + \int _{{\mathbb T}^d}\bar{\rho }\phi \Big |^{s=t}_{s=0} dx + \int _0^t\int _{{\mathbb T}^d}\bar{\rho }\nabla \bar{\rho }\cdot \nabla \phi dxds, \end{aligned}$$

32 $$\begin{aligned} 0&= -\varepsilon \int _0^t\int _{{\mathbb T}^d}\bar{\rho }\bar{u}\cdot \partial _s\psi dxds + \varepsilon \int _{{\mathbb T}^d}\bar{\rho }\bar{u}\cdot \psi \Big |^{s=t}_{s=0} dx \nonumber \\&\phantom {xx} - \varepsilon \int _0^t\int _{{\mathbb T}^d}\bar{\rho }(\bar{u}\otimes \bar{u}):\nabla \psi dxds - \varepsilon \int _0^t\int _{{\mathbb T}^d}\bar{\rho }\bar{e}\cdot \psi dxds \end{aligned}$$

for test functions $\phi \in C_0^\infty ({\mathbb T}^d\times [0,t])$ and $\psi \in C_0^\infty ({\mathbb T}^d\times [0,t];{\mathbb R}^d)$.

We derive the energy equality associated to (29)–(30). For this, we use the test function $\phi =\bar{\rho }-\varepsilon |\bar{u}|^2/2$ in (31) and recall that $\nabla \bar{\rho }=-\bar{u}$:

$$\begin{aligned} 0&= -\int _0^t\int _{{\mathbb T}^d}\bar{\rho }\partial _s\bigg (\bar{\rho }- \frac{\varepsilon }{2}|\bar{u}|^2\bigg ) dxds + \int _{{\mathbb T}^d}\bar{\rho }(t)\bigg (\bar{\rho }(t) - \frac{\varepsilon }{2} |\bar{u}(t)|^2\bigg )dx \\&\phantom {xx}- \int _{{\mathbb T}^d}\bar{\rho }^0\bigg (\bar{\rho }^0 - \frac{\varepsilon }{2}|\bar{u}^0|^2\bigg ) dx + \int _0^t\int _{{\mathbb T}^d}\bar{\rho }|\bar{u}|^2 dx ds + \varepsilon \int _0^t\int _{{\mathbb T}^d}\bar{\rho }\bar{u}\cdot \nabla \bar{u}\cdot \bar{u} dxds. \end{aligned}$$

Furthermore, with the test function $\psi =\bar{u}$ in (32), we find that

$$\begin{aligned} 0&= -\varepsilon \int _0^t\int _{{\mathbb T}^d}\bar{\rho }\bar{u}\cdot \partial _s\bar{u} dxds + \varepsilon \int _{{\mathbb T}^d}\bar{\rho }(t)|\bar{u}(t)|^2 dx - \varepsilon \int _{{\mathbb T}^d}\bar{\rho }^0|\bar{u}^0|^2 dx \\&\phantom {xx} - \varepsilon \int _0^t\int _{{\mathbb T}^d}\bar{\rho }\bar{u}\cdot \nabla \bar{u}\cdot \bar{u}dxds - \varepsilon \int _0^t\int _{{\mathbb T}^d}\bar{\rho }\bar{e}\cdot \bar{u}dxds. \end{aligned}$$

Adding the last two equations, some terms cancel, and observing that

$$ \int _0^t\int _{{\mathbb T}^d}\bar{\rho }\partial _s\bar{\rho }dxds = \frac{1}{2}\int _{{\mathbb T}^d}\bar{\rho }^2\Big |^{s=t}_{s=0}dx $$

leads to the energy equality

33 $$\begin{aligned} E_0(\bar{\rho }(t),\bar{u}(t)) - E_0(\bar{\rho }^0,\bar{u}^0) + \int _0^t\int _{{\mathbb T}^d}\bar{\rho }|\bar{u}|^2 dxds = \varepsilon \int _0^t\int _{{\mathbb T}^d}\bar{\rho }\bar{e}\cdot \bar{u} dxds, \end{aligned}$$

where the energy associated to (29)–(30) is defined by

$$\begin{aligned} E_0(\bar{\rho },\bar{u}) = \int _{{\mathbb T}^d}\bigg (\frac{\bar{\rho }^2}{2} + \frac{\varepsilon }{2}\bar{\rho }|\bar{u}|^2\bigg )dx. \end{aligned}$$

### Difference of mass balance equations

We subtract the energy equality (33) from the energy inequality (14) (recall that $k_1=k_2=1$):

34 $$\begin{aligned} E(&\rho ^\varepsilon (t),u^\varepsilon (t)-E_0(\bar{\rho }(t),\bar{u}(t)) + \int _0^t\int _{{\mathbb T}^d}\bigg (\sum _{i=1}^2\rho _i^\varepsilon |u_i^\varepsilon |^2 -\bar{\rho }|\bar{u}|^2\bigg )dxds \nonumber \\&\phantom {xx}+ \varepsilon \sum _{i=1}^2\int _0^t\int _{{\mathbb T}^d} \big (\rho _i^\varepsilon |\nabla u_i^\varepsilon |^2 + |u_i^\varepsilon |^2 + \rho _i^\varepsilon |u_i^\varepsilon |^4\big )dxds + \varepsilon \int _0^t\int _{{\mathbb T}^d}\bar{\rho }\bar{e}\cdot \bar{u}dxds \nonumber \\&\le E(\rho ^0,u^0) - E_0(\bar{\rho }^0,\bar{u}^0). \end{aligned}$$

We use the test function $\phi =\bar{\rho }-\varepsilon |\bar{u}|^2/2$ in the sum of the mass balance equations (12) over $i=1,2$ (recalling that $\bar{\rho }^\varepsilon =\rho _1^\varepsilon +\rho _2^\varepsilon$) and subtract the weak formulation (31) with the same test function (observing that the terms at $t=0$ cancel):

35 $$\begin{aligned} 0&= -\int _0^t\int _{{\mathbb T}^d}(\bar{\rho }^\varepsilon -\bar{\rho }) \partial _s\bigg (\bar{\rho }- \frac{\varepsilon }{2}|\bar{u}|^2\bigg )dxds + \int _{{\mathbb T}^d}(\bar{\rho }^\varepsilon -\bar{\rho })(t) \bigg (\bar{\rho }(t) - \frac{\varepsilon }{2}|\bar{u}(t)|^2\bigg )dx \nonumber \\&\phantom {xx}- \int _0^t\int _{{\mathbb T}^d}\bigg (\sum _{i=1}^2 \rho _i^\varepsilon u_i^\varepsilon - \bar{\rho }\bar{u}\bigg )\cdot \nabla \bigg (\bar{\rho }- \frac{\varepsilon }{2}|\bar{u}|^2\bigg )dxds. \end{aligned}$$

### Difference of momentum balance equations

We add the weak formulation (13) with test function $\psi =\bar{u}_i$ for $i=1,2$ and subtract the weak formulation (32) for the limit system with the same test function (recalling that $(\bar{\rho },\bar{u})$ is assumed to be smooth):

36 $$\begin{aligned} 0&= -\varepsilon \int _0^t\int _{{\mathbb T}^d}\bigg (\sum _{i=1}^2\rho _i^\varepsilon u_i^\varepsilon - \bar{\rho }\bar{u}\bigg )\cdot \partial _s\bar{u} dxds + \varepsilon \int _{{\mathbb T}^d}\bigg (\sum _{i=1}^2\rho _i^\varepsilon u_i^\varepsilon - \bar{\rho }\bar{u}\bigg )\cdot \bar{u}\bigg |^{s=t}_{s=0}dx\nonumber \\&\phantom {xx} -\varepsilon \int _0^t\int _{{\mathbb T}^d}\bigg (\sum _{i=1}^2\rho _i^\varepsilon u_i^\varepsilon \otimes u_i^\varepsilon - \bar{\rho }\bar{u}\otimes \bar{u}\bigg ):\nabla \bar{u}dxds \nonumber \\&\phantom {xx}+ \varepsilon \int _0^t\int _{{\mathbb T}^d}\sum _{i=1}^2 \big \{\rho _i^\varepsilon \nabla u_i^\varepsilon :\nabla \bar{u} + \Delta (\rho _i^\varepsilon )^{1/2}\big (2\nabla (\rho _i^\varepsilon )^{1/2}\cdot \bar{u} + (\rho _i^\varepsilon )^{1/2}\operatorname {div}\bar{u}\big )\big \}dxds \nonumber \\&\phantom {xx} + \varepsilon \int _0^t\int _{{\mathbb T}^d}\sum _{i=1}^2 \big (u_i^\varepsilon \cdot \bar{u} + \rho _i^\varepsilon |u_i^\varepsilon |^2 u_i^\varepsilon \cdot \bar{u}\big )dxds + \int _0^t\int _{{\mathbb T}^d}\bigg (\sum _{i=1}^2\rho _i^\varepsilon u_i^\varepsilon - \bar{\rho }\bar{u}\bigg )\cdot \bar{u} dxds \nonumber \\&\phantom {xx} + \int _0^t\int _{{\mathbb T}^d}\big (\bar{\rho }^\varepsilon \nabla \bar{\rho }^\varepsilon - \bar{\rho }\nabla \bar{\rho }\big )\cdot \bar{u} dxds + \varepsilon \int _0^t\int _{{\mathbb T}^d}\bar{\rho }\bar{e}\cdot \bar{u}dxds. \end{aligned}$$

### Relative energy inequality

We recall the definition of the relative energy:

$$\begin{aligned} E_R(\rho ^\varepsilon ,u^\varepsilon |\bar{\rho },\bar{u}) = \int _{{\mathbb T}^d}\bigg \{\frac{1}{2}(\bar{\rho }^\varepsilon -\bar{\rho })^2 + \varepsilon \sum _{i=1}^2\bigg (\frac{\rho _i^\varepsilon }{2}|u_i^\varepsilon -\bar{u}|^2 + |\nabla (\rho _i^\varepsilon )^{1/2}|^2\bigg )\bigg \}dx, \end{aligned}$$

where $\bar{\rho }^\varepsilon :=\rho _1^\varepsilon +\rho _2^\varepsilon$. The main task is to derive the relative entropy inequality.

#### Lemma 13

(Relative energy inequality) There exists a constant $C>0$ independent of $\varepsilon$ such that

$$\begin{aligned} E_R(&\rho ^\varepsilon (t),u^\varepsilon (t)|\bar{\rho }(t),\bar{u}(t)) + \int _0^t\int _{{\mathbb T}^d}\sum _{i=1}^2\rho _i^\varepsilon |u_i^\varepsilon -\bar{u}|^2 dxds \\&\phantom {xx}+ \frac{\varepsilon }{2}\int _0^t\int _{{\mathbb T}^d}\sum _{i=1}^2\big ( \rho _i^\varepsilon |\nabla (u_i^\varepsilon -\bar{u})|^2 + |u_i^\varepsilon -\bar{u}|^2 + \rho _i^\varepsilon |u_i^\varepsilon |^2|u_i^\varepsilon -\bar{u}|^2\big )dxds \\&\le C\root 4 \of {\varepsilon } + C\int _0^tE_R(\rho ^\varepsilon ,u^\varepsilon |\bar{\rho },\bar{u})(s)ds. \end{aligned}$$

In particular, for $i=1,2$, as $\varepsilon \rightarrow 0$,

$$\begin{aligned} (\rho _i^\varepsilon )^{1/2}(u_i^\varepsilon -\bar{u})\rightarrow 0 \quad \text{ strongly } \text{ in } L^2(0,T;L^2({\mathbb T}^d)). \end{aligned}$$

#### Proof

We subtract (35) and (36) from (34). An elementary computation shows that

37 $$\begin{aligned} \int _{{\mathbb T}^d}&\bigg (\frac{1}{2}(\bar{\rho }^\varepsilon -\bar{\rho })^2 + \frac{\varepsilon }{2}\sum _{i=1}^2\rho _i^\varepsilon |u_i^\varepsilon -\bar{u}|^2 + \varepsilon \sum _{i=1}^2|\nabla (\rho _i^\varepsilon )^{1/2}|^2\bigg )\Big |^{s=t}_{s=0}dx = J_1+\cdots +J_{11}, \end{aligned}$$

where

$$\begin{aligned} J_1&= -\int _0^t\int _{{\mathbb T}^d}\sum _{i=1}^2\rho _i^\varepsilon |u_i^\varepsilon -\bar{u}|^2dxds, \\ J_2&= -\int _0^t\int _{{\mathbb T}^d}\bigg (\sum _{i=1}^2\rho _i^\varepsilon u_i^\varepsilon - \bar{\rho }^\varepsilon \bar{u}\bigg )\cdot \bar{u} dxds, \\ J_3&= -\int _0^t\int _{{\mathbb T}^d}(\bar{\rho }^\varepsilon -\bar{\rho })\partial _s \bigg (\bar{\rho }- \frac{\varepsilon }{2}|\bar{u}|^2\bigg )dxds, \\ J_4&= -\varepsilon \int _0^t\int _{{\mathbb T}^d}\bigg (\sum _{i=1}^2\rho _i^\varepsilon u_i^\varepsilon - \bar{\rho }\bar{u}\bigg )\cdot \partial _s\bar{u}dxds, \\ J_5&= -\int _0^t\int _{{\mathbb T}^d}\bigg (\sum _{i=1}^2\rho _i^\varepsilon u_i^\varepsilon - \bar{\rho }\bar{u}\bigg )\cdot \nabla \bigg (\bar{\rho }- \frac{\varepsilon }{2}|\bar{u}|^2\bigg )dxds, \\ J_6&= -\varepsilon \int _0^t\int _{{\mathbb T}^d}\bigg (\sum _{i=1}^2 \rho _i^\varepsilon u_i^\varepsilon \otimes u_i^\varepsilon - \bar{\rho }\bar{u}\otimes \bar{u} \bigg ):\nabla \bar{u}dxds, \\ J_7&= \int _0^t\int _{{\mathbb T}^d}(\bar{\rho }^\varepsilon \nabla \bar{\rho }^\varepsilon - \bar{\rho }\nabla \bar{\rho })\cdot \bar{u}dxds, \\ J_8&= -\varepsilon \int _0^t\int _{{\mathbb T}^d}\sum _{i=1}^2\rho _i^\varepsilon \nabla u_i^\varepsilon :\nabla (u_i^\varepsilon -\bar{u})dxds, \\ J_9&= -\varepsilon \int _0^t\int _{{\mathbb T}^d}\sum _{i=1}^2 u_i^\varepsilon \cdot (u_i^\varepsilon -\bar{u})dxds, \\ J_{10}&= -\varepsilon \int _0^t\int _{{\mathbb T}^d}\sum _{i=1}^2\rho _i^\varepsilon |u_i^\varepsilon |^2 u_i^\varepsilon \cdot (u_i^\varepsilon -\bar{u})dxds, \\ J_{11}&= \varepsilon \int _0^t\int _{{\mathbb T}^d}\Delta (\rho _i^\varepsilon )^{1/2} \big (2\nabla (\rho _i^\varepsilon )^{1/2}\cdot \bar{u} + (\rho _i^\varepsilon )^{1/2} \operatorname {div}\bar{u}\big )dxds. \end{aligned}$$

The expression on the left-hand side of (37) at $t=0$ is of order $\varepsilon$, because of Assumption (A2) on the initial data and $(\rho ^\varepsilon -\bar{\rho })(0)=0$. We wish to estimate $J_1,\ldots ,J_{11}$.

We split the sum $J_3+\cdots +J_6$ into two parts, $J_3+\cdots +J_6=K_1+K_2$, where

$$\begin{aligned} K_1&= \frac{\varepsilon }{2}\int _0^t\int _{{\mathbb T}^d}(\bar{\rho }^\varepsilon -\bar{\rho }) \partial _s|\bar{u}|^2 dxds - \varepsilon \int _0^t\int _{{\mathbb T}^d}\bigg (\sum _{i=1}^2\rho _i^\varepsilon u_i^\varepsilon - \bar{\rho }\bar{u}\bigg )\cdot \partial _s\bar{u}dxds \\&\phantom {xx}+ \frac{\varepsilon }{2}\int _0^t\int _{{\mathbb T}^d} \bigg (\sum _{i=1}^2\rho _i^\varepsilon u_i^\varepsilon - \bar{\rho }\bar{u}\bigg ) \nabla |\bar{u}|^2 dxds \\&\phantom {xx} - \varepsilon \int _0^t\int _{{\mathbb T}^d}\bigg (\sum _{i=1}^2\rho _i^\varepsilon u_i^\varepsilon \otimes u_i^\varepsilon - \bar{\rho }\bar{u}\otimes \bar{u}\bigg ) :\nabla \bar{u} dxds \\&=: K_{11}+\cdots K_{14}, \\ K_2&= -\int _0^t\int _{{\mathbb T}^d}(\bar{\rho }^\varepsilon -\bar{\rho })\partial _s\bar{\rho }dxds - \int _0^t\int _{{\mathbb T}^d}\bigg (\sum _{i=1}^2\rho _i^\varepsilon u_i^\varepsilon - \bar{\rho }\bar{u}\bigg )\cdot \nabla \bar{\rho }dxds. \end{aligned}$$

Some terms cancel in $K_{11}+K_{12}$, and we end up with

$$\begin{aligned} K_{11}+K_{12} = -\varepsilon \int _0^t\int _{{\mathbb T}^d}\bigg (\sum _{i=1}^2 \rho _i^\varepsilon u_i^\varepsilon - \bar{\rho }^\varepsilon \bar{u}\bigg )\cdot \partial _s\bar{u}dxds. \end{aligned}$$

Also in the sum $K_{13}+K_{14}$, some terms cancel:

$$\begin{aligned} K_{13}+K_{14}&= -\varepsilon \int _0^t\int _{{\mathbb T}^d}\bigg (\sum _{i=1}^2 \rho _i^\varepsilon u_i^\varepsilon - \bar{\rho }^\varepsilon \bar{u}\bigg )\cdot \nabla \bar{u} \cdot \bar{u}dxds \\&\phantom {xx}- \varepsilon \int _0^t\int _{{\mathbb T}^d}\sum _{i=1}^2 \rho _i^\varepsilon (u_i^\varepsilon -\bar{u}) \otimes (u_i^\varepsilon -\bar{u}):\nabla \bar{u}dxds. \end{aligned}$$

Recalling that $\bar{e}=\partial _t\bar{u}+\bar{u}\cdot \nabla \bar{u}$, we obtain

$$\begin{aligned} K_1&= -\varepsilon \int _0^t\int _{{\mathbb T}^d}\bigg (\sum _{i=1}^2 \rho _i^\varepsilon u_i^\varepsilon - \bar{\rho }^\varepsilon \bar{u}\bigg )\cdot \bar{e} dxds \\&\phantom {xx}- \varepsilon \int _0^t\int _{{\mathbb T}^d}\sum _{i=1}^2 \rho _i^\varepsilon (u_i^\varepsilon -\bar{u}) \otimes (u_i^\varepsilon -\bar{u}):\nabla \bar{u}dxds. \end{aligned}$$

Since $\bar{e}$ and $\bar{u}$ are assumed to be smooth, we can estimate $K_1$ according to

$$\begin{aligned} K_1&\le \varepsilon C(\bar{e})\Vert \rho _1^\varepsilon u_1^\varepsilon + \rho _2^\varepsilon u_2^\varepsilon - \bar{\rho }^\varepsilon \bar{u}\Vert _{L^2(0,T;L^1({\mathbb T}^d))} \\&\phantom {xx}+ \varepsilon C(\nabla \bar{u})\int _0^t\int _{{\mathbb T}^d}\rho _i^\varepsilon |u_i^\varepsilon -\bar{u}|^2 dxds \le C\varepsilon , \end{aligned}$$

where the last step follows from estimates (27).

For $K_2$, we insert $\partial _t\bar{\rho }=-\operatorname {div}(\bar{\rho }\bar{u})$, use $\nabla \bar{\rho }=-\bar{u}$, and integrate by parts:

$$\begin{aligned} K_2&= -\int _0^t\int _{{\mathbb T}^d}\nabla (\bar{\rho }^\varepsilon -\bar{\rho }) \cdot (\bar{\rho }\bar{u})dxds + \int _0^t\int _{{\mathbb T}^d} \bigg (\sum _{i=1}^2\rho _i^\varepsilon u_i^\varepsilon - \bar{\rho }\bar{u}\bigg ) \cdot \bar{u}dxds. \end{aligned}$$

We add to this expression

$$\begin{aligned} J_2+J_7&= -\int _0^t\int _{{\mathbb T}^d}\bigg (\sum _{i=1}^2 \rho _i^\varepsilon u_i^\varepsilon - \bar{\rho }^\varepsilon \bar{u}\bigg )\cdot \bar{u} dxds + \int _0^t\int _{{\mathbb T}^d}(\bar{\rho }^\varepsilon \nabla \bar{\rho }^\varepsilon - \bar{\rho }\nabla \bar{\rho })\cdot \bar{u} dxds, \end{aligned}$$

which yields, using again $\bar{u}=-\nabla \bar{\rho }$ and integration by parts,

$$\begin{aligned} K_2 + J_2 + J_7&= -\int _0^t\int _{{\mathbb T}^d}\big (\bar{\rho }(\nabla \bar{\rho }^\varepsilon -\nabla \bar{\rho }) + (\bar{\rho }\bar{u}-\bar{\rho }^\varepsilon \bar{u}) - (\bar{\rho }^\varepsilon \nabla \bar{\rho }^\varepsilon - \bar{\rho }\nabla \bar{\rho }) \big )\cdot \bar{u} dxds \\&= \int _0^t\int _{{\mathbb T}^d}(\bar{\rho }^\varepsilon -\bar{\rho }) \nabla (\bar{\rho }^\varepsilon -\bar{\rho })\cdot \bar{u} dxds = -\frac{1}{2}\int _0^t\int _{{\mathbb T}^d}(\bar{\rho }^\varepsilon -\bar{\rho })^2 \operatorname {div}\bar{u}dxds \\&\le C(\bar{u})\int _0^t\int _{{\mathbb T}^d}(\bar{\rho }^\varepsilon -\bar{\rho })^2 dxds. \end{aligned}$$

This shows that

$$\begin{aligned} J_2+\cdots +J_7 = K_1 + K_2 + J_2 + J_7 \le C\varepsilon + C\int _0^t\int _{{\mathbb T}^d}(\bar{\rho }^\varepsilon -\bar{\rho })^2 dxds. \end{aligned}$$

Next, we estimate $J_8$, using the Young and Cauchy–Schwarz inequalities:

$$\begin{aligned} J_8&= -\varepsilon \int _0^t\int _{{\mathbb T}^d}\sum _{i=1}^2 \big (\rho _i^\varepsilon |\nabla (u_i^\varepsilon -\bar{u})|^2 + \rho _i^\varepsilon \nabla \bar{u}:\nabla (u_i^\varepsilon -\bar{u})\big )dxds \\&\le -\frac{\varepsilon }{2}\int _0^t\int _{{\mathbb T}^d}\sum _{i=1}^2 \rho _i^\varepsilon |\nabla (u_i^\varepsilon -\bar{u})|^2 dxds + \varepsilon \sum _{i=1}^2\Vert \rho _i^\varepsilon \Vert _{L^\infty (0,T;L^1({\mathbb T}^d))} \Vert \nabla \bar{u}\Vert _{L^2(0,T;L^\infty ({\mathbb T}^d))}^2 \\&\le -\frac{\varepsilon }{2}\int _0^t\int _{{\mathbb T}^d}\sum _{i=1}^2 \rho _i^\varepsilon |\nabla (u_i^\varepsilon -\bar{u})|^2 dxds + C\varepsilon , \end{aligned}$$

since the total mass of $\rho _i^\varepsilon$ is uniformly bounded. We estimate $J_9$ and $J_{10}$ in a similar way, leading to

$$\begin{aligned} J_9 + J_{10}&\le -\frac{\varepsilon }{2}\int _0^t\int _{{\mathbb T}^d}\sum _{i=1}^2\big ( |u_i^\varepsilon -\bar{u}|^2 + \rho _i^\varepsilon |u_i^\varepsilon |^2|u_i^\varepsilon -\bar{u}|^2 \big )dxds + C\varepsilon . \end{aligned}$$

By the uniform estimates (27),

$$\begin{aligned} J_{11}&\le \root 4 \of {\varepsilon }\sum _{i=1}^2\sqrt{\varepsilon } \Vert \Delta (\rho _i^\varepsilon )^{1/2}\Vert _{L^2(0,T;L^2({\mathbb T}^d))}\big (4\root 4 \of {\varepsilon } \Vert \nabla (\rho _i^\varepsilon )^{1/4}\Vert _{L^4(0,T;L^4({\mathbb T}^d))} \\&\phantom {xx}\times \Vert (\rho _i^\varepsilon )^{1/4}\Vert _{L^4(0,T;L^4({\mathbb T}^d))} \Vert \bar{u}\Vert _{L^\infty (0,T;L^\infty ({\mathbb T}^d))} \\&\phantom {xx}+ \Vert (\rho _i^\varepsilon )^{1/2}\Vert _{L^\infty (0,T;L^2({\mathbb T}^d))} \Vert \operatorname {div}\bar{u}\Vert _{L^2(0,T;L^\infty ({\mathbb T}^d))}\big ) \le C(\bar{u})\root 4 \of {\varepsilon }. \end{aligned}$$

Thus, we obtain

$$\begin{aligned} J_8&+\cdots +J_{11} \\&\le C\root 4 \of {\varepsilon } - \frac{\varepsilon }{2} \int _0^t\int _{{\mathbb T}^d}\sum _{i=1}^2\big ( \rho _i^\varepsilon |\nabla (u_i^\varepsilon -\bar{u})|^2 + |u_i^\varepsilon -\bar{u}|^2 + \rho _i^\varepsilon |u_i^\varepsilon |^2|u_i^\varepsilon -\bar{u}|^2\big )dxds. \end{aligned}$$

We summarize the previous estimates to infer from (37) that

$$\begin{aligned} \int _{{\mathbb T}^d}&\bigg (\frac{1}{2}(\rho ^\varepsilon -\bar{\rho })^2 + \frac{\varepsilon }{2}\sum _{i=1}^2\rho _i^\varepsilon |u_i^\varepsilon -\bar{u}|^2 + \varepsilon \sum _{i=1}^2|\nabla (\rho _i^\varepsilon )^{1/2}|^2\bigg )(t)dx \\&\phantom {xx} + \int _0^t\int _{{\mathbb T}^d}\sum _{i=1}^2\rho _i^\varepsilon |u_i^\varepsilon -\bar{u}|^2 dxds \\&\phantom {xx}+ \frac{\varepsilon }{2}\int _0^t\int _{{\mathbb T}^d}\sum _{i=1}^2\big ( \rho _i^\varepsilon |\nabla (u_i^\varepsilon -\bar{u})|^2 + |u_i^\varepsilon -\bar{u}|^2 + \rho _i^\varepsilon |u_i^\varepsilon |^2|u_i^\varepsilon -\bar{u}|^2\big )dxds \\&\le C\root 4 \of {\varepsilon } + C\int _0^t\int _{{\mathbb T}^d} (\bar{\rho }^\varepsilon -\bar{\rho })^2 dxds. \end{aligned}$$

Gronwall’s lemma concludes the proof of the lemma. $\square$

### Proof of Theorem 3

It follows from Lemma 13 that

$$\begin{aligned} \Vert \rho _i^\varepsilon (u_i^\varepsilon -\bar{u})\Vert _{L^1(0,T;L^1({\mathbb T}^d))} \le \Vert (\rho _i^\varepsilon )^{1/2}\Vert _{L^2(0,T;L^2({\mathbb T}^d))} \Vert (\rho _i^\varepsilon )^{1/2}(u_i^\varepsilon -\bar{u})\Vert _{L^2(0,T;L^2({\mathbb T}^d))} \rightarrow 0 \end{aligned}$$

as $\varepsilon \rightarrow 0$. Together with the convergence $\rho _i^\varepsilon \rightharpoonup \rho _i$ weakly* in $L^\infty (0,T;L^2({\mathbb T}^d))$, this shows that

$$\begin{aligned} \rho _i^\varepsilon u_i^\varepsilon = \rho _i^\varepsilon (u_i^\varepsilon -\bar{u}) + \rho _i^\varepsilon \bar{u} \rightharpoonup \rho _i\bar{u} \quad \text{ weakly } \text{ in } L^1(0,T;L^1({\mathbb T}^d)). \end{aligned}$$

By definition, $\bar{u}=-\nabla \bar{\rho }$. Since $\rho _i^\varepsilon u_i^\varepsilon \rightharpoonup J_i$ weakly in $L^2(0,T;L^{4/3}({\mathbb T}^d))$, we infer that $J_i=-\rho _i\nabla \bar{\rho }$. In particular, $\rho _i$ solves the transport equation $\partial _t\rho _i=-\operatorname {div}J_i = \operatorname {div}(\rho _i\nabla \bar{\rho })$, while $\bar{\rho }$ is the solution to the porous-medium equation $\partial _t\bar{\rho }=\operatorname {div}(\bar{\rho }\nabla \bar{\rho })$ with $\bar{\rho }(0)=\rho _1^0+\rho _2^0$ in ${\mathbb T}^d$.

## Data Availability

There is no data associated to this work.
